# MIiSR: Molecular Interactions in Super-Resolution Imaging Enables the Analysis of Protein Interactions, Dynamics and Formation of Multi-protein Structures

**DOI:** 10.1371/journal.pcbi.1004634

**Published:** 2015-12-11

**Authors:** Fabiana A. Caetano, Brennan S. Dirk, Joshua H. K. Tam, P. Craig Cavanagh, Maria Goiko, Stephen S. G. Ferguson, Stephen H. Pasternak, Jimmy D. Dikeakos, John R. de Bruyn, Bryan Heit

**Affiliations:** 1 The J. Allyn Taylor Centre for Cell Biology, Robarts Research Institute and the Department of Physiology and Pharmacology, The University of Western Ontario, London, Ontario, Canada; 2 Department of Microbiology and Immunology, The University of Western Ontario, London, Ontario, Canada; 3 Department of Physics and Astronomy, The University of Western Ontario, London, Ontario, Canada; 4 Cellular and Molecular Medicine, University of Ottawa, Ottawa, Ontario, Canada; 5 Department of Clinical Neurological Sciences, Schulich School of Medicine, The University of Western Ontario, London, Ontario, Canada; Johns Hopkins University, UNITED STATES

## Abstract

Our current understanding of the molecular mechanisms which regulate cellular processes such as vesicular trafficking has been enabled by conventional biochemical and microscopy techniques. However, these methods often obscure the heterogeneity of the cellular environment, thus precluding a quantitative assessment of the molecular interactions regulating these processes. Herein, we present Molecular Interactions in Super Resolution (MIiSR) software which provides quantitative analysis tools for use with super-resolution images. MIiSR combines multiple tools for analyzing intermolecular interactions, molecular clustering and image segmentation. These tools enable quantification, in the native environment of the cell, of molecular interactions and the formation of higher-order molecular complexes. The capabilities and limitations of these analytical tools are demonstrated using both modeled data and examples derived from the vesicular trafficking system, thereby providing an established and validated experimental workflow capable of quantitatively assessing molecular interactions and molecular complex formation within the heterogeneous environment of the cell.

## Author Summary

In this paper we present the software package Molecular Interactions in Super Resolution (MIiSR), which provides a series of quantitative analytical tools for measuring molecular interactions and the formation of higher-order molecular complexes in super-resolution microscopy images.

“This is a *PLOS Computational Biology* Methods paper.”

## Introduction

The regulation of many biological processes requires exquisite control over the formation of multimolecular complexes in specific regions of the cell. For example, the endocytosis, transport and exocytosis phases of vesicular trafficking require the coordination of multiple regulatory proteins, signaling lipids and second messengers on the plasma membrane, endoplasmic reticulum, Golgi and multiple other vesicular structures (reviewed in [1–9]). Importantly, in these systems a single protein can evoke different responses depending on its subcellular localization and the interacting partners present in a particular cellular niche. This complexity is well illustrated by the Phosphofurin Acidic Cluster Sorting Protein 2 (PACS-2) which regulates ER-mitochondria traffic through interactions with BAP31 [10], cytosol-to-mitochondria and cytosol-to-lysosome translocation of apoptotic effectors through interactions with Bid, Bim and Bax [10,11], induces cell cycle arrest through interacting with nuclear-localized SIRT1 [12], and is even targeted by pathogens such as HIV to misdirect MHC I endocytosed from the cell surface [13]. Adding further complexity is the requirement that these proteins be scaffolded into high-order structures such as receptor complexes, coated pits and membrane microdomains to mediate their function [14–17]. While this building of molecularly unique complexes enables a small number of regulatory proteins to coordinate a vast and heterogeneous vesicular trafficking system, the commonly employed molecular and biochemical approaches used to identify and characterize these complexes obscures the heterogeneity that provides specificity to these cellular systems. For example, the *en bulk* nature of biochemical assays such as immunoprecipitation do not preserve the heterogeneity and subcellular localization of these systems, while conventional microscopy-based assays suffer from limited spatial resolution (typically 250–350 nm [18]). Indeed, many tools have been developed to overcome these limitations of optical microscopy, including Forster Resonance Energy Transfer (FRET) and Bimolecular Fluorescence Complementation (BiFC) [19,20]. While these technologies have provided an increased understanding of the unique molecular complexes that regulate cellular processes such as vesicular trafficking, they typically involve energy transfer between two fluorophores, or reconstitution of fluorophore halves, and thus are usually limited to assessing interactions between two protein species [21], can suffer from low signal-to-noise ratios (FRET, [22]) and may alter protein interaction dynamics through irreversible cross-linking (BiFC, [23]). Super-resolution microscopy avoids these issues by directly imaging fluorophores with high precision, with the number of molecular interactions that can be measured limited only by the number of available fluorescent channels.

The advent of super-resolution microscopy systems has enabled the direct visualization of individual molecular complexes with high precision [24,25], with improvements in resolution achieved by one of two strategies. The first strategy uses patterned excitation light that confines fluorophore excitation to sub-resolution regions (e.g. STimulated Emission Depletion Microscopy (STED, [26,27]) and Saturated Structured Illumination Microscopy (SSIM [28])). The second strategy relies on the stochastic switching of fluorophores between dark and fluoresce states at low densities, followed by localization of each individual fluorophore by mapping a Gaussian function to each fluorophore’s point-spread function (e.g. Photoactivation Localization Microscopy (PALM, [29–31]) and Ground State Depletion Microscopy (GSDM, [32]). Although the stochastic techniques generally provide higher lateral (*xy*) resolution (20–30 nm, [31–33]) than do structured illumination methods (50–80 nm, [26–28]), both methods provide sufficient resolution to quantify intermolecular interactions. Recent advances such as the use of astigmatic lenses have provided similar improvements in axial (*z*) resolution (50–60 nm), allowing quantification to be extended into three dimensions. Several of these methods can be deployed using widely available laser-based Total Internal Reflection Fluorescence (TIRF) microscopes and free software [34], allowing super-resolution microscopy to be easily and inexpensively implemented in many existing microscopy facilities. Although a powerful tool, super-resolution microscopy lacks a standardized set of validated quantitative tools for assessing intermolecular interactions equivalent to the commonly employed colocalization and morphological methods available to conventional microscopy [18,35]. Indeed, many analytical techniques developed for conventional microscopy produce erroneous results if applied to super-resolution images, due both to super-resolution images with pixels smaller than the size of many of the complexes being imaged–leading to interacting molecules being resolved in neighboring (non-colocalizing) pixels–and due to the stochastic nature of super-resolution imaging which can lead simultaneously to both missed fluorophore detections (undersampling) and repeat detection of fluorophores (oversampling) within the same sample [36–38].

To address these limitations we have developed Molecular Interactions in Super-Resolution (MIiSR), a validated set of software tools for quantifying intermolecular interactions and the formation of higher-order molecular complexes in super-resolution images. MIiSR overcomes the issues associated with applying conventional colocalization and morphological analyses to super-resolution images through the use of spatial statistical approaches which elucidate the presence of intermolecular interactions and the presence of large molecular assemblies through quantification of the spatial relationship between labeled molecules. By taking a statistical approach, MIiSR provides precise quantitative data from super-resolution images, even given the issues of oversampling, undersampling, and differential sampling between channels, and allows these analyses to be applied to the molecular position files produced by most super-resolution imaging systems.

## Design & Implementation

MIiSR is comprised of a set of analytical functions and two graphical user interfaces (GUI’s) written in the Matlab mathematical language (S1 –MIiSR Program). The input required by MIiSR are molecular position files produced by many super-resolution microscopy systems. MIiSR natively supports positions files from Leica GSD-SR Ground-State Depletion microscopes, Zeiss ELYRA PS1 dSTORM microscopes, and any microscope running QuickPALM software [34]. In addition, the import of multiple formats of tab-delineated positions files is also supported, allowing for import from many other super-resolution microscopy systems. Best analysis practices require data files containing the X/Y/Z coordinates of the detected molecules, as well as the number of photons collected or precision of detection, for each detected molecule–although data lacking Z coordinates and/or photon/precision data can be analyzed. To install, decompress the MIiSR.zip file and add the resulting “MIiSR” folder to the path file in Matlab.

**Fig 1:**
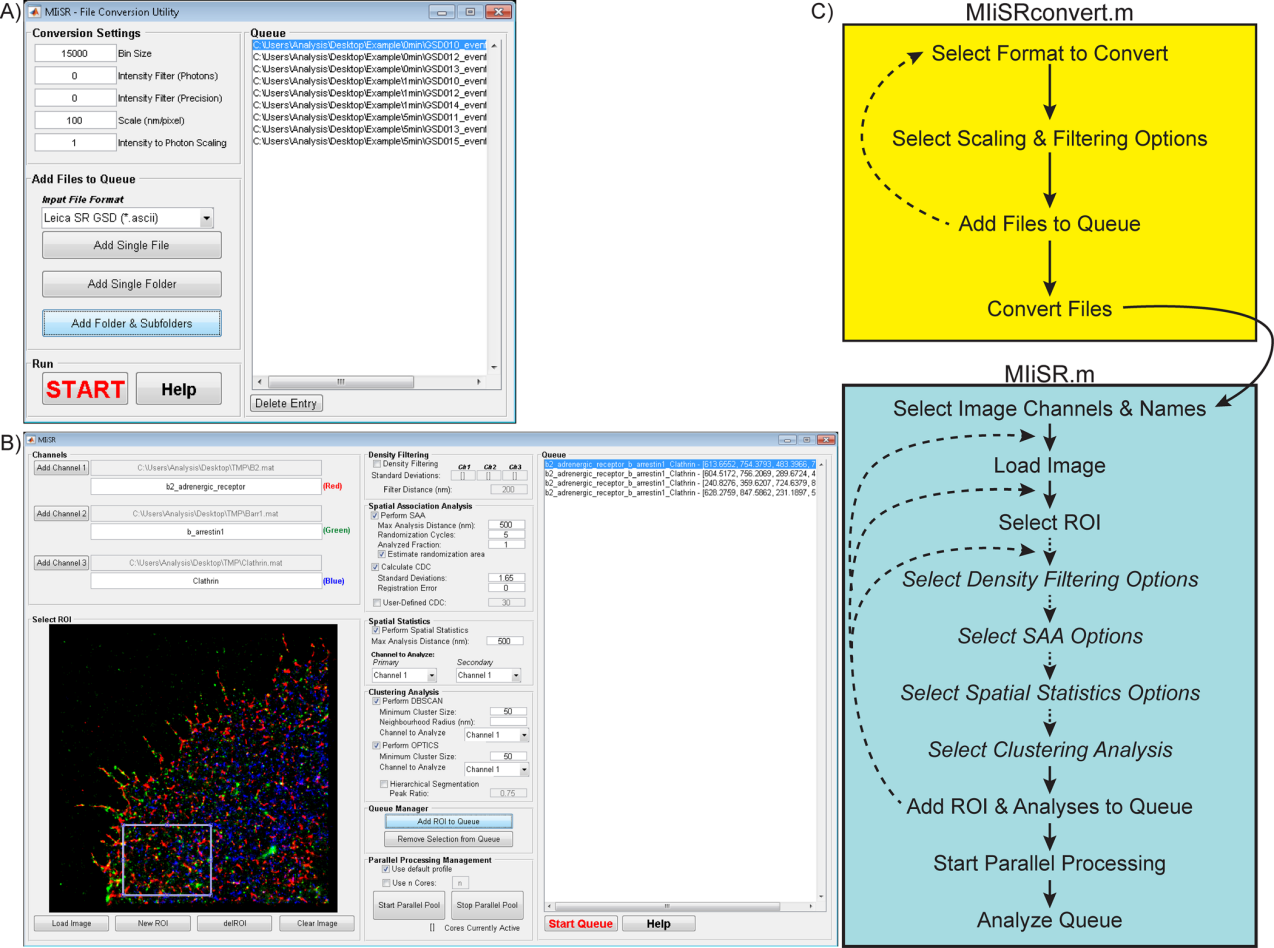
MIiSR graphical user interfaces and work flow. (A) Image of the MIiSRconvert GUI, which is used to convert microscope position files from a manufacturer’s format into a MIiSR-compatible format. A complete description of use of the GUI can be found in its help file. The example GUI has had multiple Leica SR GSD position files loaded into the queue for processing. (B) Image of the MIiSR GUI, which is used to perform the analyses described in this paper on super-resolution position files generated by MIiSRconvert. Four ROIs from the current image have been added to the queue. A complete description of the use of the GUI can be found in its help file, and all assays are described in this paper. (C) Workflow of MIiSR analysis. Solid arrows indicate mandatory steps, dotted arrows and *italicized text* indicate optional steps.

The first GUI, run using the command ‘MIiSRconvert’, enables the batch conversion of microscope position files to Matlab-compatible.mat files in a standardized format (X coordinate, Y coordinate, Z coordinate, # Photons, Precision of detection, Fig 1A). Files can be added to the conversion queue individually, by folder, or by folder and all sub-folders. Options are available to scale molecular positions from pixels to nm, to scale intensity to number of detected photons, to filter out poorly resolved fluorophores based on fluorophore intensity or the precision of detection, and to optimize processing speed. The integrity of the molecule position data is maintained through applying these scaling factors in a linear fashion to all data points in the position file. Once processing has been started all files in the queue will be converted, with converted files saved in the same folder as the original position file. MIiSRconvert acts as a GUI for the function fileConv.m, which can operate independently of the GUI, enabling its use in user-written Matlab functions and scripts.

The second GUI, run using the command ‘MIiSR’, is the primary interface for analysis of super-resolution images (Fig 1B). This GUI acts as a wrapper for functions which combine color (image) channels and provide image cropping (LoadCrop.m), filter out molecules located in low-density regions in order to enhance analysis of clustered molecules (densityFilter.m), quantify intermolecular interactions (SAA2col.m and SAA3col.m), quantify molecular clustering (spatialStats.m), and segment molecular clusters within super-resolution images (DBSCAN.m, OPTICS.m and hierOPTICS.m). These functions can operate independently of the GUI, allowing for their incorporation into user-written functions and scripts. The general workflow for MIiSR (Fig 1C) is to load one to three color channels, which will generate a preview image, and using this preview, to select a Region Of Interest (ROI) to analyze. Once an ROI is set, the user configures the analyses they wish to perform, and then adds the ROI and its attendant analysis options to the queue. The user then has four options–the analysis conditions for the current ROI can be changed and the new analysis scheme saved as a new entry in the queue, a new ROI and analysis scheme can be selected from the existing image and added to the queue, a new image can be loaded and ROIs and analysis schemes from the new image added to the queue, and lastly, the queue can be processed (Fig 1C). During processing a folder will be generated for each entry in the queue, located in the same folder as the position file for the first (red) color channel. The data, graphs and images generated for each ROI will be saved in this folder.

While the analysis routines provided in the MIiSR GUI represent a series of well-validated and powerful analytical tools, alternative methods to perform similar analyses have been published previously. To provide a comprehensive set of tools, we have included functions for Owen *et al’s*. density-based method of cluster identification (Hsegment.m) and Sengupta *et al’s* pair correlation based analysis of cluster composition (RDFquant.m) [15,39]. These command-line functions perform these analysis on the molecular position files produced by the MIiSR GUI, and can be run as either stand-alone functions or can be incorporated into user-written scripts and functions. All functions provided in the MIiSR package are extensively documented through the use of in-function comments, enabling users to modify each function as needed.

## Results

### Detection of Inter-protein Interactions with Spatial Association Analysis

Detecting intermolecular interactions, in the context of their subcellular localization, is the aim of many biochemical fractionation and imaging methods. Super-resolution imaging offers an unprecedented opportunity to investigate intermolecular interactions, as many super resolution microscopy methods can detect fluorophores with a lateral resolution of ~20 nm, which is sufficiently accurate to enable a statistical assessment of intermolecular distances [31–33]. By comparing distances between the molecule of interest and its nearest neighbor(s) in the other imaging channels, statistical evidence of inter-molecular interactions can be established. Indeed, we have previously used this nearest-neighbor approach to establish that tetraspanin’s scaffold the formation of molecularly distinct CD36-integrin complexes, an observation that was obscured by conventional imaging and biochemical assays [40]. This toolbox includes a more powerful form of this Spatial Association Assay (SAA), which begins with a conventional nearest-neighbor approach wherein the Euclidian distance from each molecule in one color channel of the image to its nearest neighbor(s) in the other color channel(s) (S1A Fig), but rather than defining intermolecular interactions as those occurring below an arbitrarily selected threshold, SAA instead introduces a statistically robust detection of intermolecular interactions by defining potentially interacting molecules as those separated by distances below the Colocalization Distance Criterion (CDC). The fraction of interactions is then compared to a randomized image to determine if the observed degree of interaction is above those predicted of non-interacting samples, thus determining if the observed interactions are real or due to chance juxtaposition of molecules in the image (S1A Fig). The CDC is defined by two factors, the precision of fluorophore detection, and by any chromatic registration defects in the microscope’s optical path. The precision of localization of each detected molecule in an image is usually provided in the position files produced by super-resolution microscopes, while any registration errors must be measured directly [41], but are generally negligible in most commercial super-resolution microscopy systems. The CDC is defined as the root-mean-squared error of the sum of mean precision across color channels (σ_RMS_), multiplied by a 90% or 95% probability cutoff (1.65 or 2 standard deviations), with any image registration (I_reg_) error added to this product:

$\sigma_{RMS}={\left(\sum_{i=1}^{n}\;\sigma_{ci}\right)}^{\frac{1}{2}}$, where σ_ci_ is the mean precision of color channel *i*.

$$CDC=1.65\sigma_{RMS}+I_{reg}$$

Thus the CDC represents a statistically defined maximum separation distance between interacting molecules in a super-resolution image after sampling precision and chromatic aberration are taken into account (S1 Text, [42]). For 2-color images most microscopes will have a CDC of 23–28 nm, the exact value of which is determined by the numerical aperture and magnification of the objective lens, and the brightness and number of fluorophores detected in each color channel; for 3-color images the CDC is larger, due to the increased degree of error incurred by measuring across an additional color channel, with typical values ranging from 28 to 35 nm. It is important to note that SAA analysis is highly sensitive to the area selected for analysis; ROIs which extend past the boarders of the cell, or which include unlabeled structures that have displaced the labeled molecules within the ROI, can lead to over-estimation of the degree of molecular interactions during the randomization process–specifically, leading to the randomization of molecular positions over a larger area/volume than that truly occupied by the imaged molecules. To limit this error, the user should ensure that all ROIs used for SAA analyses fall within the boundaries of the cell and lack any obvious voids. In addition, MIiSR includes the option to estimate the true area occupied by the labeled molecules, through refining the user-selected ROI to a minimum bounding polygon encompassing all points in the original ROI. Proper selection of ROIs, along with this automated refinement of the randomization area, will ensure accurate quantification of molecular interactions in super-resolution images.

**Fig 2:**
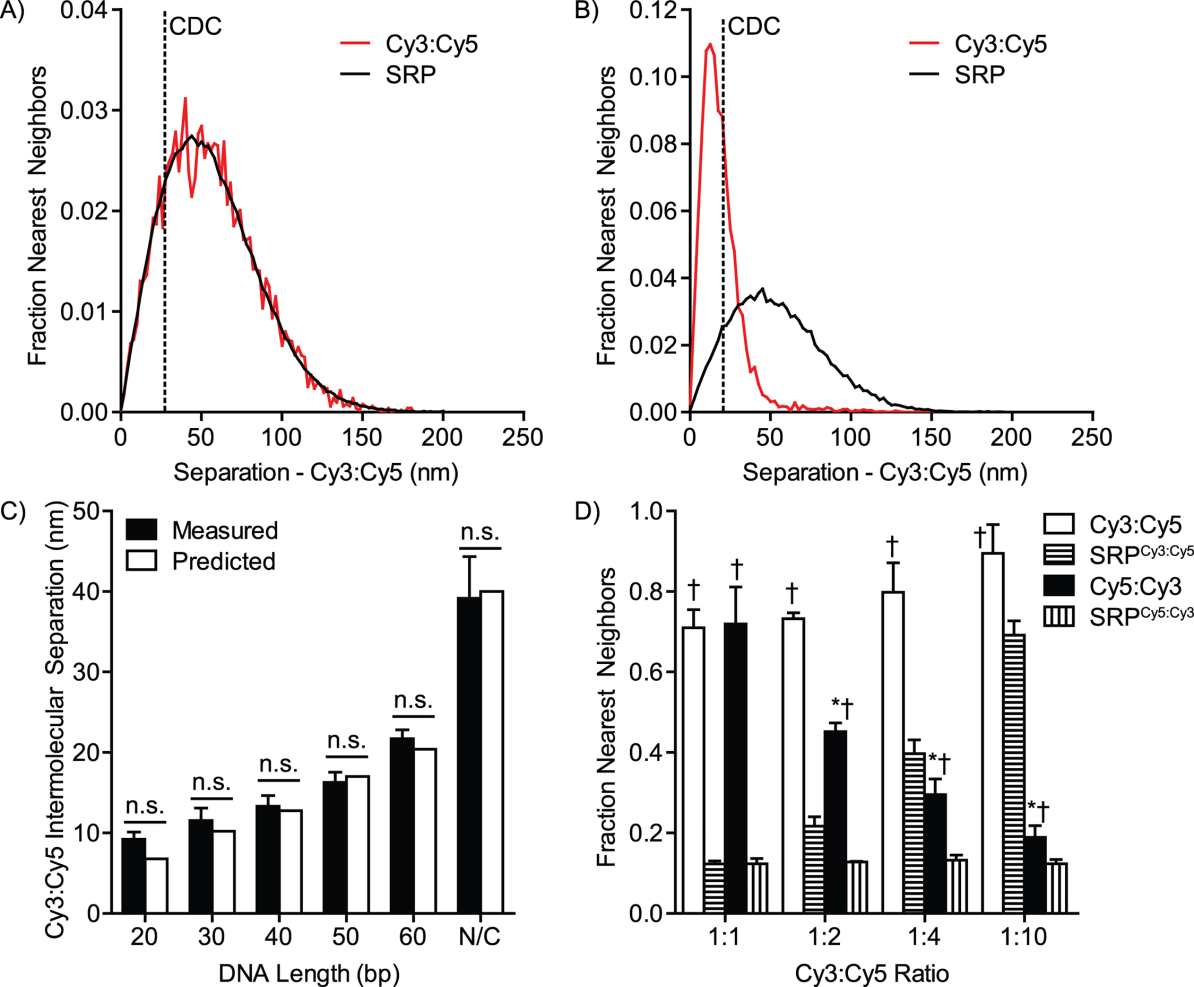
Validation of Spatial Association Analysis (SAA) using defined-length DNA oligomers. (A) SAA histogram plotting the distance between non-complementary 30-mer DNA oligomers 5’ labeled with Cy3 and Cy5. The vertical dotted line indicates the Colocalization Distance Criterion (CDC), the separation distance below which molecules are potentially interacting. To determine if the degree of colocalization is significant the positions of the Cy5-labeled molecules were randomized using a Monte Carlo approach and the SAA measurement repeated (SRP). (B) SAA histogram plotting the distance between annealed, complementary 30-mer DNA oligomers 5’ labeled with Cy3 and Cy5. (C) Measured and predicted intermolecular distances between Cy3 and Cy5 on differing length complementary DNA oligos 5’ labeled with Cy3 and Cy5 deposited on coverslips at equimolar concentration. N/C indicates the measured distance of non-complementary Cy3 and Cy5 labeled oligos deposited at the same concentration. (D) Impact of partial intermolecular interactions on SAA analysis, determined by annealing complementary 30-mer DNA oligomers 5’ labeled with Cy3 and Cy5 at differing ratios. SRP = Simulated Random Positions. A-B: Representative histograms, C-D: Data are presented as mean ± SEM. n = 3, minimum of 5 images per experiment. n.s. = not significantly different, paired *t-*test, † = p < 0.05 compared to SRP, * = p < 0.05 compared to Cy5:Cy3 at a 1:1 ratio.

To demonstrate the efficacy of SAA analysis we generated 30-mer complementary and non-complementary DNA oligos 5’ tagged with either Cy3 or Cy5 (Fig 2). Annealed complementary oligos create Cy3/Cy5 pairs separated by 10.2 nm [43], while the non-complementary oligos distribute randomly. As expected, MIiSR analysis of 1:1 mixtures of non-complementary oligos found no significant association, and displayed a distribution of intermolecular distances identical to that measured in randomized images (Fig 2A). In contrast, there was a marked increase in intermolecular separations below the CDC in a 1:1 mixture of complementary oligos, with the mode of the distribution curve falling close to the predicted 10.2 nm separation of the Cy3 and Cy5 fluorophores (Fig 2B). Indeed, measurement of intermolecular distances of Cy3/Cy5 labeled complementary DNA oligos ranging from 20 bp to 60 bp (estimated lengths of 6.8 to 20.4 nm [43]), determined that the mode of the SAA plot accurately measured the predicted intermolecular distances, and could provide an accurate measure of intermolecular distances as small as one-third the precision of the super-resolution microscope (Fig 2C). It is important to note that the calculation of interactions by SAA is non-symmetrical, meaning that the degree of association observed between molecule A and molecule B will be different from that measured between B and A. This is illustrated when complementary Cy3 and Cy5 labeled oligos are mixed such that there is excess Cy5-labeled oligo, leading to a mixture containing annealed Cy3-Cy5 oligos and non-annealed Cy5 oligos (Fig 2D). As all Cy3 oligos are bound to a Cy5 oligo, the SAA interaction observed between Cy3 and Cy5 remains constant, whereas increasing the portion of unannealed Cy5 oligo decreased the measured interaction of the Cy5 oligo with the Cy3 oligo (Fig 2D). MIiSR automatically calculates SAA along all lines of symmetry, providing a full assessment of molecular interactions within an image. In theory, the statistical approach taken in SAA analysis should render it insensitive to modest over- and under-sampling. To test this we imaged complementary Cy3 and Cy5 oligos and then modified the data sets to mimic over- and under-sampling. Modest oversampling had a minimal impact on the observed degree of colocalization (S1B Fig), whereas under-sampling reduced the measured degree of colocalization (S1C Fig). Importantly, however, undersampling did not change the measured colocalization when normalized to the colocalization observed in randomized images (S1D Fig), indicating that image randomization can be used to normalize for differing degrees of sampling. It is important to note that because of oversampling it is not practical to use SAA analysis to quantify homeotypic interactions as it is extremely difficult to differentiate between the repeated detection of the same fluorophore versus detection of multiple fluorophores within an area less than the CDC (S1E Fig). Moreover, extreme oversampling can lead to randomized images where the mean intermolecular separation is less than the CDC. For highly oversampled images, MIiSR includes the option to analyze a randomly selected subset of the molecules in the image, a method demonstrated previously to provide accurate quantification of intermolecular interactions [40].

**Fig 3:**
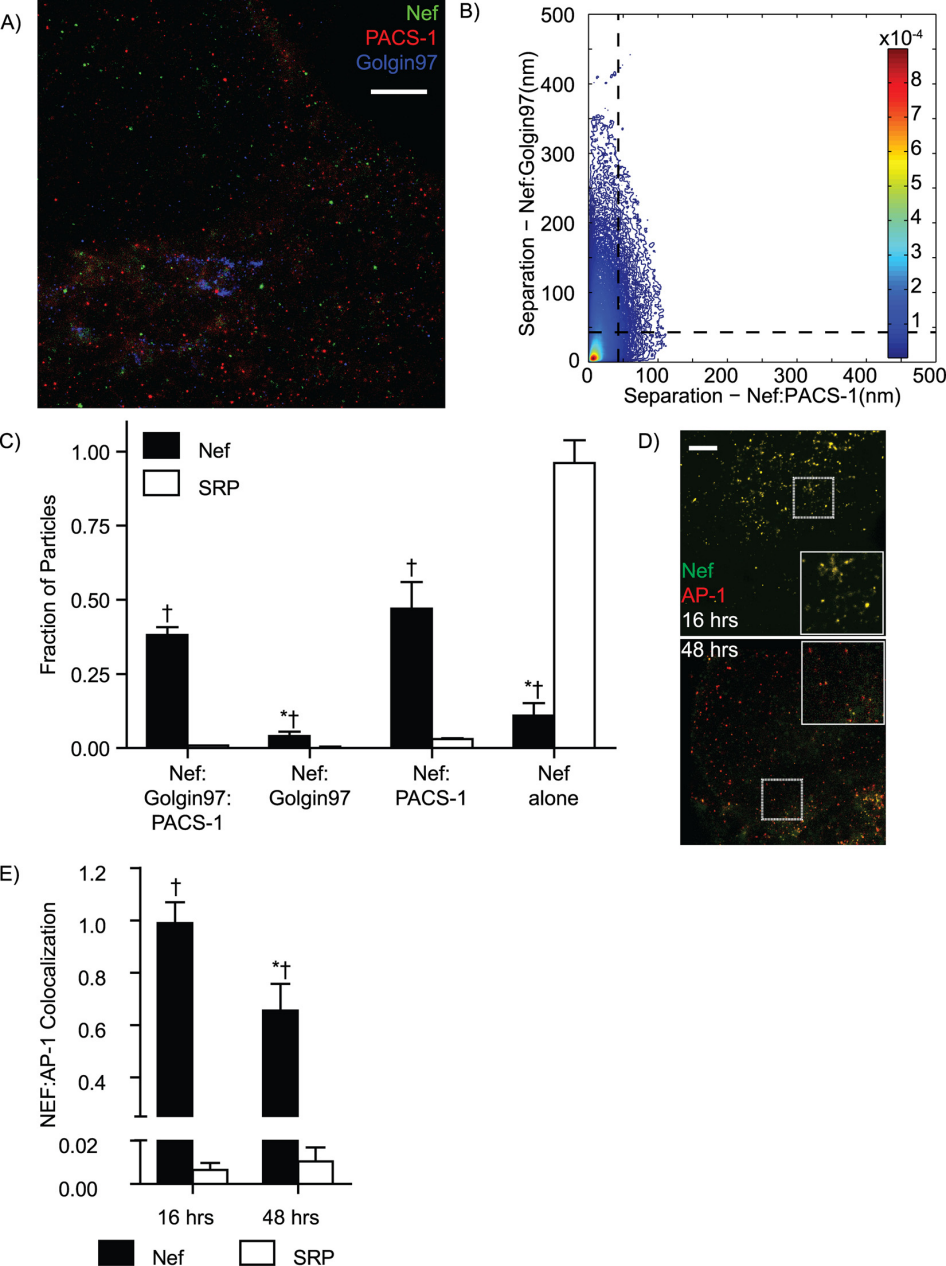
Quantification of intermolecular interactions between Nef, PACS-1, AP-1 and the Golgi, using Spatial Association Analysis. (A) Representative image of GFP-Nef, PACS-1-mCherry and Golgin97 staining in a CD4+ HeLa cell 48 hours post-transfection. (B) SAA histogram of panel A, plotting the distance between each Nef molecule and the nearest PACS-1 and nearest Golgin97. Horizontal and vertical dotted lines indicate the CDC below which molecules are potentially colocalized. (C) Quantification of Nef colocalization patterns with Golgin97 and PACS-1, with the fraction of Nef colocalized with both Golgin97 and PACS-1, associated with either Golgin97 or PACS-1, or not colocalized with either molecule indicated. (D) Images of and (E) quantification of AP-1 and Nef colocalization patterns during the endocytosis-targeting (16 hrs) and Golgi-targeting (48 hrs) phases of Nef activity. SRP = Simulated Random Positions. A,D are representative images of, and B,C,E quantify 3 experiments, 5 images per experiment. Data are presented as mean ± SEM, † = p < 0.05 compared to SRP, * p < 0.05 compared to NEF-Golgin97-PACS-1 (C) or 16 hrs (E). Scale bars 2.5 μm, inserts are 3.5 μm x 3.5 μm.

To demonstrate the utility of SAA analysis in a biologically relevant system we used MIiSR to analyze interactions with the HIV protein Nef, which modulates the trafficking regulators PACS-1 and AP-1 in order to alter membrane trafficking in a manner advantageous to HIV immune evasion [13,44]. Using cells expressing GFP-Nef, mCherry-PACS-1 and Alexa-647 immunolabled Golgin 97, we used GSDM to image the interaction of Nef with PACS-1 and the Golgi, and then analyzed these images using 3-color SAA analysis (Fig 3A and 3B). This analysis revealed that the majority of Nef is complexed with PACS-1, with a third of the Nef-PACS-1 complexes localized to the Golgi (Fig 3B and 3C). The small portion of non-PACS-1 associated Nef was split between Golgi and non-Golgi compartments, and importantly, all of these interactions were significantly different from the degree of association predicted for non-interacting molecules (Fig 3C, SRP). By fixing and imaging cells at different time points, SAA analysis can be applied to the study of temporal changes in protein-protein interactions. Indeed, we found that HIV Nef decreases its interaction with the trafficking regulator PACS-1 as time increases after HIV protein expression (Fig 3D and 3E), consistent with the shift of Nef’s function from altering endocytosis to altering Golgi export over this time period [45]. Clearly, MIiSR can provide insight into intermolecular interactions not otherwise detectable by conventional biochemical and microscopy techniques.

### Quantifying Molecular Clustering

**Fig 4:**
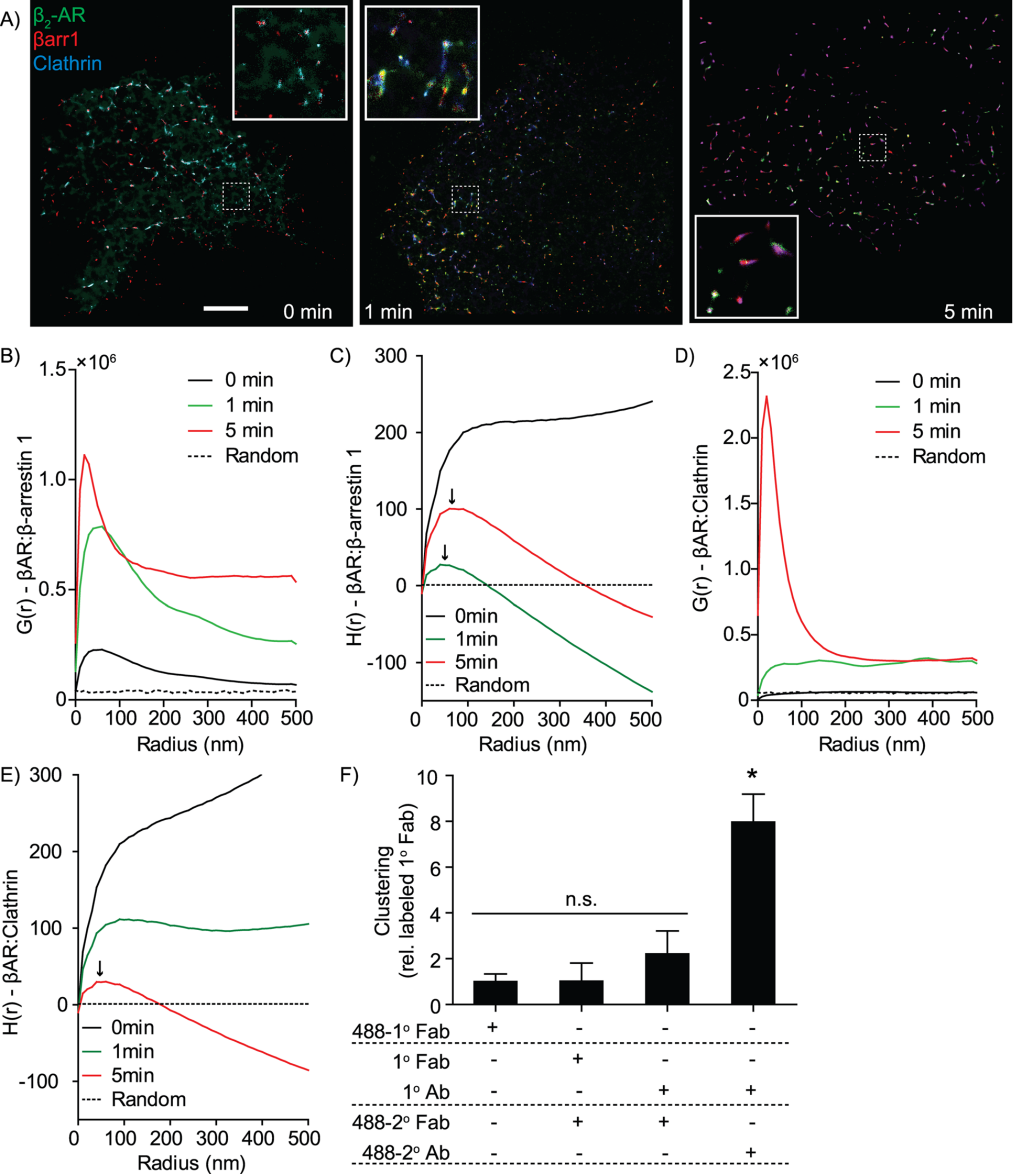
Quantifying co-clustering using the radial distribution function and Ripley’s K function. (A) The β_2_-adrenergic receptor (β_2_-AR, Green), β-arrestin-1 (βarr1, Red) and Clathrin (Blue) form an internalization complex after stimulation of the β_2_-adrenergic receptor in HEK293 cells with 10 μM isoproterenol. (B-C) Quantification of co-clustering of the β_2_-adrenergic receptor with β-arrestin-1 by (D) RDF analysis and (E) Ripley’s H-function, before (0 min) and 1 and 5 minutes post-stimulation with isoproterenol. (D-E) Quantification of β_2_-adrenergic receptor co-clustering with clathrin coated pits by (D) RDF analysis and (E) Ripley’s H-function, before (0 min) and 1 and 5 minutes post-stimulation with isoproterenol. (F) Self-clustering of the tetraspanin CD81 induced by the use of polyvalent versus mono/divalent labeling reagents. Ab = antibody. All images are representative of, and graphs quantify, 3 experiments with a minimum of 5 images per experiment. Data are presented as mean ± SEM, * = p < 0.05, n.s. = not significantly different, ANOVA with Bonferroni correction. Scale bar (A) is 2.5 μm, inserts are 1.47 μm x 1.47 μm. D-E: Arrows indicate mean cluster size, as determined by the radius of maximum H(r).

While SAA quantification provides insights into intermolecular interactions, it does not provide information on the formation of higher-order molecular structures such as protein islands, lipid microdomains, vesicles, or other large molecular assemblies. These higher-order structures are critical in many biological systems, for example, comprising the 90 nm diameter endocytic clathrin-coated pits [17]. Indeed, GSDM imaging of the β_2_-adrenergic receptor after stimulation with 10 μM isoproterenol shows a visually apparent increase in co-clustering with β-arrestin-1 and clathrin (Fig 4A), consistent with our previous studies showing this receptor to be internalized via a β-arrestin-1/clathrin-dependent pathway [46,47]. While formation of *de novo* interactions of the β_2_-adrenergic receptor with β-arrestin-1 and clathrin can be identified by SAA, the delivery of β_2_-adrenergic receptor to clathrin-coated pits by β-arrestin-1 is best assessed using clustering assays. Quantification of molecular clustering is implemented in MIiSR using spatial statistics, specifically the Radial Distribution Function (RDF) and Ripley’s K statistic [48–50]. These analyses provide quantifiable, numerical measures of clustering that can be compared across multiple images.

RDF, also known as the pair-correlation function and G-function, was developed nearly 50 years ago for the assessment of atomic and molecular distributions in physical and chemical systems [51], and more recently has been applied to super-resolution images [15,38,48]. RDF quantifies the density of molecules as a function of distance (r) to other molecules in the same color channel (self-clustering), or in a second (co-clustering), color channel (S2A Fig and S1 Text). RDF analysis will accurately illustrate the presence of multiple cluster sizes and inter-cluster distance (S2A Fig), and the relative degree of clustering is indicated by the height of the peaks corresponding to the molecular clusters. An alternative approach proposed by Ripley in 1977, termed the K function, quantifies the number of molecules as a function of distance (S3A Fig and S1 Text, [52]). The K function is typically normalized to distance and density, producing the H function. Ripley’s H function, plotted against intermolecular distance (r), produces an inverted parabola-like curve with a single peak. The value of r corresponding to this peak (r_max_) correlates roughly with mean cluster size, while the height of this peak correlates to the degree of clustering in the sample. It is important to note that the value of r_max_ is impacted by the distance between neighboring clusters, and thus is not a direct indicator of mean cluster size [50,53]. Several approaches are available to calculate an accurate mean cluster radius from H-plots; Lagache *et al*, determined that average cluster radius could be approximated by r_max_/1.3 in biological systems [53], whereas Kiskowski *et al*, determined that the mean cluster radius was equal to one-half the radius where the derivative of the H(r) plot [H’(r)] crosses -1 [50]. MIiSR calculates both H(r) and H’(r), allowing the user to use either method to determine mean cluster radius. The difference between Ripley’s H-function and RDF analysis is illustrated in S1–S3 Videos, which respectively compare: S1 Video) the co-clustering of two randomly distributed molecular species, S2 Video) co-clustering of a randomly distributed population of molecules with a pre-clustered population of molecules, and S3 Video) the impact of increasing ratios of unclustered to clustered molecules on these analyses. Of note, RDF analysis is better able to identify weak co-clustering (S1 and S2 Videos), and retains its ability to identify clusters in the presence of a large number of unclustered molecules (S3 Video). In contrast, Ripley’s analysis provides a single clear peak indicative of mean cluster size, whereas RDF produces a more complex, multipeaked curve, indicative of sub-populations of differently sized clusters (S1–S3 Videos).

The utility of these tools in MIiSR is demonstrated by analyzing the sequential formation of a macromolecular endocytic complex formed by the β_2_-adrenergic receptor, its endocytic regulator β-arrestin-1, and clathrin-coated pits (Fig 4A). Following stimulation with isoproterenol the β_2_-adrenergic receptor associates with β-arrestin-1, which subsequently delivers the β-adrenergic receptor to clathrin-coated pits for endocytosis [46,54,55]. RDF analysis revealed that unstimulated β_2_-adrenergic receptor is minimally clustered with β-arrestin-1 (Fig 4B), with this weak clustering indicated in Ripley’s analysis as an upward curve (Fig 4C). As expected, stimulation with isoproterenol results in a rapid co-clustering of the β-adrenergic receptor with β-arrestin-1 that increases over time (Fig 4A–4C). In contrast, no significant clustering of the β-adrenergic receptor is observed with clathrin prior to, or in the first minute following, isoproterenol stimulation, with significant co-clustering between the β-adrenergic receptor and clathrin observed only at 5 minutes post-stimulation (Fig 4A, 4D and 4E). Interestingly, the β_2_-adrenergic receptor clusters start off as small clusters (~40 nm, Fig 4D, 1 minute arrow) and then coalesced into larger structures at 5 minutes (~90 nm, Fig 4D, 5 minute arrow). These larger clusters overlap, and are of the same size (90 nm) as, the β_2_-adrenergic receptor/clathrin clusters observed at the same time point (5 minute arrows, Fig 4D and 4E), and closely match the reported size of clathrin coated pits [56].

While these are powerful tools for quantifying clustering, careful sample preparation and imaging acquisition are required to minimize artifacts in both RDF and Ripley’s analysis. It is critical to employ labeling methods which minimize labeling-induced clustering. Indeed, significant antibody-induced oligomerization of CD81 was observed when samples were labeled using full-length primary and secondary antibodies, whereas replacing either the primary or secondary antibody with Fab fragments reduced CD81 oligomerization to that observed when a monomeric primary labeled Fab was used (Fig 4F). In addition, while MIiSR allows for quantification of self-clustering using both Ripley’s and RDF analyses, great caution needs to be taken when interpreting these results as oversampling caused by the repeat detection of the same fluorophores can create an artefact of intense self-clustering, in which case the apparent cluster size will equal the precision of the Gaussian mapping algorithm (S2B and S3B Figs). As a result, analysis of self-clustering remains difficult [38], although alternate methods such as Sengupta *et al*.*’s* method for quantifying cluster composition in RDF plots can provide some indication of self-clustering [15]. This function is provided as ‘RDFquant.m’ in the MIiSR package. A final consideration is image acquisition, specifically the degree of under- or over-sampling in the analyzed images. In contrast to measurements of self-clustering, neither over- or under-sampling have a significant impact on cross-RDF analysis (S2D Fig). In contrast, cross-Ripley’s analysis is sensitive to both oversampling and undersampling, with undersampling leading to underestimation of clustering, and oversampling leading to smaller measured cluster size (S3C–S3F Fig). As such, it is important that Ripley’s analysis be performed on a sample containing the smallest possible degree of over- and under-sampling. At this time there are no widely accepted methods for determining the ideal degree of sampling for quantitative super-resolution imaging, and as such we would recommend that both RDF and Ripley’s analysis be performed in parallel. Alternatively, we have determined that it is possible to define a reasonable stop-point in the image reconstruction process at which reconstructed image achieves the best balance between undersampling and oversampling (S3D–S3F Fig). By performing a Pearson’s autocorrelation as the super-resolution image is reconstructed it is possible to determine the point where the adding additional frames to the image reconstruction process ceases to add additional information to the resulting super-resolution image (S3D and S3E Fig). Autocorrelation increases rapidly early during image reconstruction as new fluorophores are added to the image, but will asymptotically approach 1 later in the reconstruction process as new fluorophore detections decrease and repeat detections increase (S3E Fig). Ripley’s analysis of a modeled super-resolution acquisitions incorporating both over- and under-sampling demonstrates that the measured H(r) values most closely match the true H(r) values when super-resolution image reconstruction is restricted to the portion of the acquisition where the autocorrelation R value is ≤ 0.990 (S3F Fig). Unfortunately, not all super-resolution systems provide positions files compatible with this form of image reconstruction, and as such this reconstruction method could not be included in MIiSR.

### Identifying Molecular Clusters

**Fig 5:**
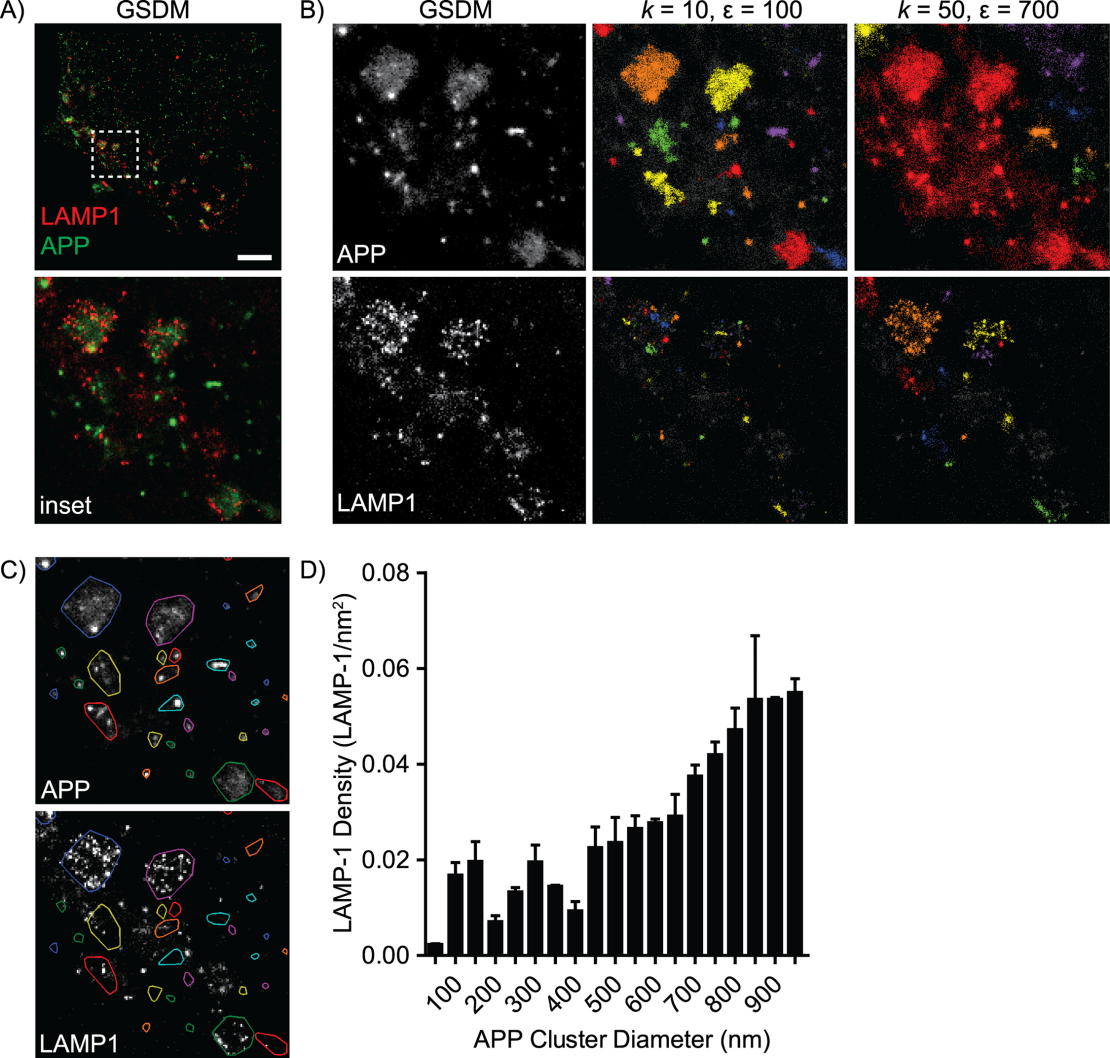
Quantification of the lysosomal marker LAMP1 in APP vesicles using the DBSCAN algorithm. (A) GSDM image analyzed in panels B-D. All analyses are performed on the region within the insert. (B) Identification of clusters using the DBSCAN algorithm using two different values for minimum cluster size (*k*) and neighbourhood size (ε), with individual clusters identified by color. (C) Top: Regions of Interest (ROIs) defined by the edge points of APP clusters identified using DBSCAN values of *k* = 10 and ε = 100; individual clusters are identified by color. Bottom: APP-defined ROIs overlaid on LAMP1 staining. (D) Quantification of LAMP1 density inside of APP clusters versus APP cluster sizes. Images are representative of, and graph quantifies, images from 3 experiments with a minimum of 4 images per experiment. Data is presented as mean ± SEM.

While the RDF and Ripley’s functions provide a quantitative measure of the degree of molecule clustering in an image, they do not allow for easy segmentation of individual clusters within the image. A previous approach, which calculates Ripley’s H function at a static value of r followed by thresholding of the resulting image, has been successful [39], and is provided in MIiSR as Hsegment.m. However, the resulting cluster map is highly dependent on the value of r and the threshold value selected by the user. As such, MIiSR also includes the cluster-identification algorithms *Density-Based Spatial Clustering of Applications with Noise* (DBSCAN, [57]) and *Ordering Points To Identify the Clustering Structure* (OPTICS, [58,59]), which are unbiased image segmentation tools for identifying molecular clusters. To demonstrate the utility and limitations of these tools they were applied to the analysis of Alzheimer’s-disease associated Amyloid Precursor Protein (APP) in lysosomes. APP may be processed into pathological forms of β-amyloid in lysosomes, with subsequent exocytosis depositing APP in the brain, thus forming the pathological β-amyloid plaques typical of Alzheimer’s disease [60–65]. The mechanism of APP release into the extracellular environment is not clear, but our earlier studies suggest that multi-vesicular body (MVB) exocytosis may be a key route of APP secretion [63,65,66]. GSDM imaging was used to identify immunolabeled APP and the lysosomal network through identifying clusters of the late endosome/lysosomal marker LAMP1 (Fig 5A). DBSCAN and OPTICS analysis in MIiSR were then applied to assess the localization of APP within the late endosome/lysosome network.

DBSCAN is an algorithmic approach to identifying clustered molecules in which a molecule is considered part of a cluster if it has a sufficient number of neighboring molecules within a defined area (S1 Text and S4A Fig, [57,67]). Unlike many clustering algorithms, DBSCAN can identify core versus edge molecules within a cluster, but is limited in that the user must choose appropriate values for neighborhood size (ε) and minimum number of molecules per cluster (*k*). Indeed, the values selected for these variables are critical, as inappropriate values can lead to over- or under-estimation of cluster composition (Fig 5B and S4B Fig). Applied properly, DBSCAN allowed for the identification of APP clusters with only minimal errors, but was unable to identify LAMP1 clusters due to the more heterogeneous nature of LAMP1 staining (Fig 5B). Regions of interest were defined using the edge molecules for each APP cluster (Fig 5C); and the density of LAMP1 within these ROIs was then quantified, demonstrating that 52.8% ± 9.7% of LAMP1 colocalized with APP clusters. Moreover, comparing the size of APP clusters to the density of LAMP1 within individual clusters revealed that large APP vesicles were LAMP1-positive, while smaller APP vesicles had minimal amounts of LAMP1 (Fig 5D). Combined, these results suggest that APP traffics through at least two different components of the endomembrane system–a LAMP1-negative compartment comprised of small vesicles, and a LAMP1-positive (lysosomal) compartment comprised of larger vesicles. Although DBSCAN was able to identify APP clusters and enabled some analysis of the distribution of APP relative to LAMP1, its inability to segment the more heterogeneous LAMP1 staining precluded analysis of APP within the lysosomal network. Indeed, this limitation of DBSCAN with super-resolution images has been noted previously, and extensive alteration of the algorithm was required to overcome these limitations, perhaps explaining why this otherwise simple algorithm has not been more widely adopted [24].

**Fig 6:**
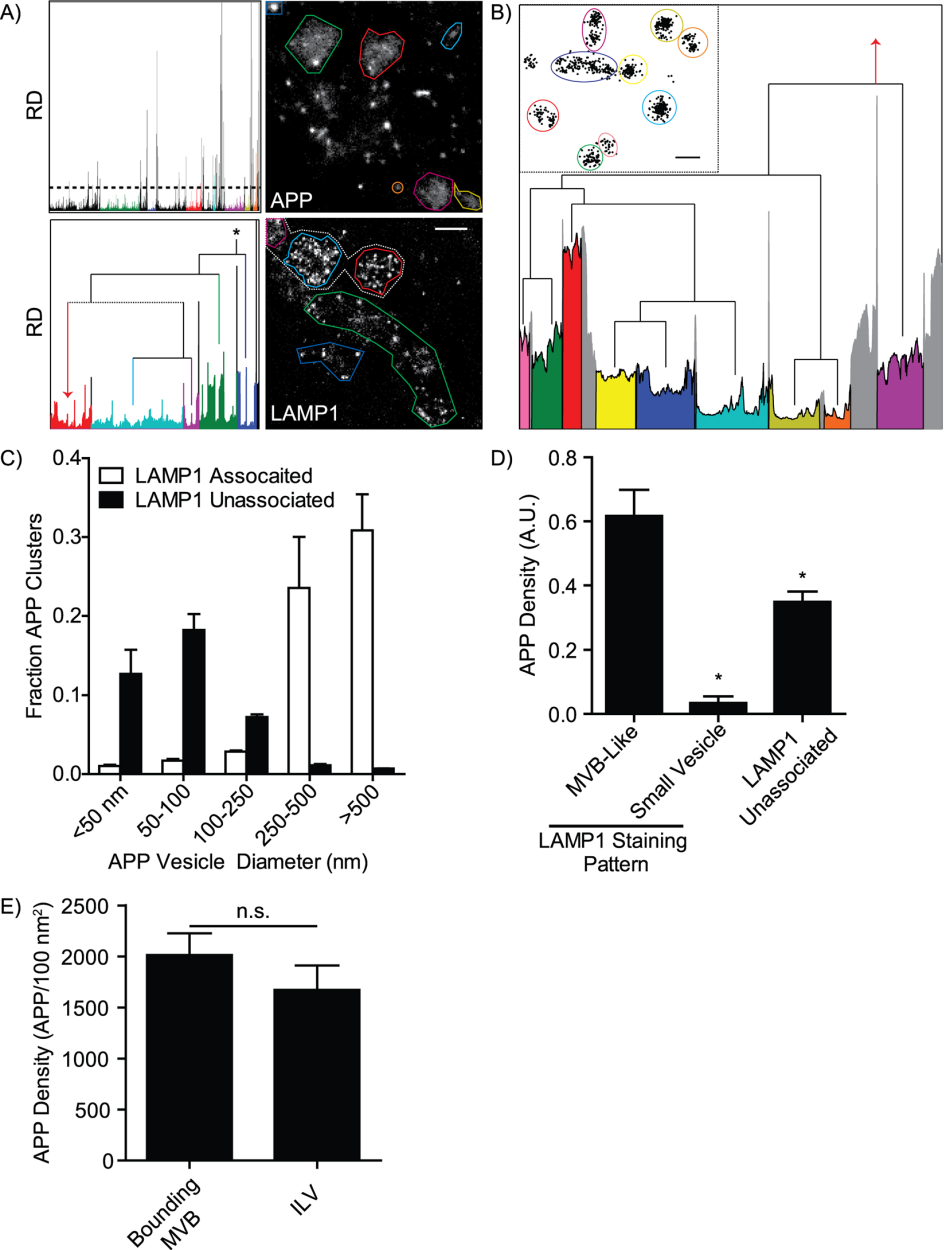
Identification of lysosomes and multivesicular bodies using the OPTICS algorithm. (A) Top: APP clusters were identified as “valleys” between RD “peaks” exceeding a static RD value (dotted line), and Bottom: LAMP1 clusters identified using a hierarchal segmentation approach. Select clusters are highlighted by colored region of the RD plots (left) and are circled by equivalent colored lines in the GSDM images (right). (B) A full hierarchal segmentation of the red LAMP1 cluster from panel A, demonstrating the nested hierarchal structure typical of multivesicular bodies. Insert plots the position of individual LAMP1 molecules within the cluster; colored circles correspond to putative intraluminal vesicles identified by the equivalent colored regions in the segmented RD plot, red arrow indicates where the RD plot joins the larger RD plot from panel A. (C) Comparison of APP cluster size and the association of APP clusters with LAMP1. (D) Quantification of APP density in LAMP1 clusters identified by OPTICS and separated based on whether the APP was associated with MVB-like, small lysosomal, or LAMP1-negative vesicles. (E) Quantification of APP density in the total (Bounding MVB) and intraluminal vesicle (ILV) portions of the MVB-like LAMP1 clusters. All images are representative of three experiments, a minimum of 4 images per experiment. Data are presented as mean ± SEM, scale bars are 500 nm (A) and 150 nm (B). * = p < 0.05 compared to MVB-like, n.s. = not significantly different.

OPTICS overcomes many of the limitations of DBSCAN and requires that the user specify only the minimum number of molecules per cluster [58,59]. Despite this, to our knowledge OPTICS analysis has not yet been employed in super-resolution images. Rather than defining clusters as regions of sufficiently high density, OPTICS instead measures the local density around each molecule and plots this density as a reachability distance (RD) plot (S1 Text and S4C–S4E Fig). Clusters appear as “valleys” in the resulting RD plot (Fig 6A and S4D Fig), with cluster identification performed visually or via automated methods [58,59,68]. When cluster density is consistent across an image a static RD value can be used to extract clusters, with clusters defined as “valleys” of points between peaks that exceed the RD cutoff (Fig 6A, APP). Static RD values do not work for cluster identification in images containing clusters of varying molecular density, as the RD value of the “valleys” is a product of a clusters molecular density (Fig 6A, LAMP1). MIiSR includes a modified version of the cluster identification method developed by Sander *et al*. (S1 Text, hierOPTICS.m, [68]) for identifying clusters in a heterogeneous population. Instead of relying on a static RD value, this method instead identifies clusters by hierarchically segmenting the RD plot at “peaks” in the plot; the plot is recursively segmented, starting with the highest peak in each segment, until a resulting segment (cluster) either contains fewer than a user-defined number of molecules, or has a mean RD value that is not sufficiently different from the peak RD value to be considered a separate feature (LAMP1 in Fig 6A and S1 Text). This method allows for identification of clusters of varying density (and thus varying RD values), for example, enabling identification of the LAMP1 clusters that could not be detected using DBSCAN (Fig 5B versus Fig 6A). This hierarchal breakdown of the RD plot also enabled identification of complex structures, for example, sub-clusters of LAMP1 within larger LAMP1 bodies–structures reminiscent of MVBs (Fig 6B). Indeed, this analysis identified numerous small LAMP1 structures 40–80 nm in diameter, the expected size of intraluminal vesicles (ILVs, [2,69,70]) organized into larger structures, 200–500 nm in diameter, consistent with the expected size of MVB’s (Fig 6A and 6B, [70]). When applied to APP, OPTICS analysis identified a similar pattern to DBSCAN, with ~60% of APP found in LAMP1 colocalizing clusters 250–800 nm in diameter, and the remainder of APP concentrated in small LAMP1-negative bodies (Fig 6C). Interestingly, the bulk of LAMP1-associated APP was found in MVB-like bodies (Fig 6D). Comparison of APP density within larger MVB-like bodies and their constituent ILV-like bodies revealed no difference in the density of APP, suggesting that APP is most likely in the lumen of MVBs and not within the lumen of ILVs (Fig 6E).

While beyond the scope of this article, many other analyses are possible on clusters identified by either DBSCAN or OPTICS including morphological analyses or generation of 3D cell models with super-resolution precision using volumetric rendering of the edges and vertices exported by MIiSR. As with RDF and Ripley’s analysis, the repeat identification of fluorophores combined with the precision of the detection algorithm tends to create the appearance of self-clustering. As such, care must be taken to exclude any clusters near or below the precision of the microscope when performing DBSCAN or OPTICS analysis.

### Combining Methods for Detailed Molecular Analysis

**Fig 7:**
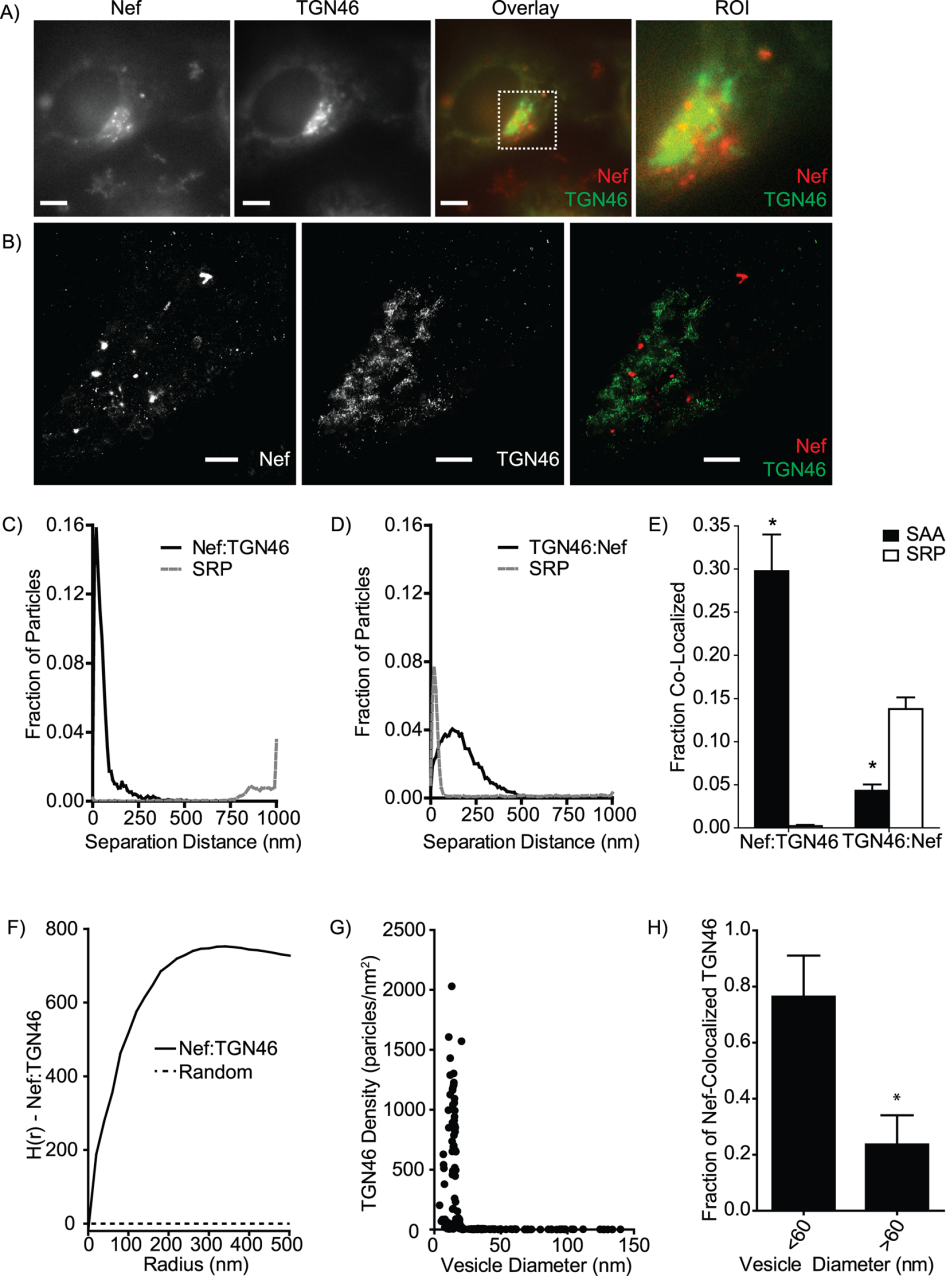
Combining multiple analyses to characterize inter-protein interactions. (A) Conventional microscopy images of dimerized Nef, detected by bimolecular fluorescence complementation (Nef, red) and the Golgi trafficking regulator TGN46 (green). ROI has a Mander’s colocalization ratio (Nef:TGN46) of 0.842. (B) GSDM image of the ROI indicated in panel A. (C-D) SAA plots and (E) quantification of Nef interactions with TGN46 (C, E) and the reverse quantification of TGN46 interaction with Nef (D,E). (F) Ripley’s H function analysis of Nef:TGN46 co-clustering. (G) TGN46 density in Nef-containing vesicles of increasing size. Nef-containing vesicles were identified using the OPTICS algorithm, and the density of TGN46 in each Nef-containing vesicle quantified. (H) Fraction of total detected TGN46 in small (<60 nm) and large (>60 nm) Nef vesicles. Images are representative of, and graphs quantify, 8 images collected in two experiments. Data are presented as mean ± SEM (E,H) or are representative plots from single images (C,D,F,G). Scale bars are 2.5 μm (A) or 0.5 μm (B), ROI (A) is 3.94 μm x 3.94 μm. SRP = Simulated Random Positions. * = p<0.05 compared to SRP, paired *t*-test (E) or vesicles <60nm, Students *t*-test (H).

In the preceeding sections we have demonstrated individual capabilities of MIiSR using examples drawn from various intracellular trafficking processes. Each method provided quantification of a single aspect of molecular interactions or higher-order structuring. However, by using MIiSR to apply a combination of these methods to a single image, a highly detailed view of the structure and organization of a molecular system can be revealed. Moreover, these quantitative image analysis tools can be combined with other readouts of molecular function, such as the identification of active signaling complexes with BiFC, to further delineate the functional aspects of a molecular system. For example, the HIV-1 protein Nef modulates vesicular trafficking in order to promote HIV survival by preventing HIV antigen bearing MHC I from remaining on the cell surface [13,44]. While Nef can form homodimers, no labeling reagents exist which selectively labels dimerized Nef; rather, this conformation can be identified using BiFC-tagged Nef [13,71]. In this assay two Nef fusion proteins, consisting of Nef fused to either the C or N terminal fragment of Venus, were co-expressed in cells; upon Nef dimerization these non-fluorescent fluorophore fragments can reconstitute a functional Venus which can then be imaged using super-resolution microscopy. Using GSDM we quantified the localization of dimerized Nef relative to the Golgi transport regulator TGN46. Mander’s colocalization analysis [72] of a conventional microscopy image indicated that there that was ~80% overlap between TGN46 and Nef-BiFC (Fig 7A). However, it is unclear if this reflects a true interaction between subsets of dimerized Nef and TGN46 or if this is due to the low spatial resolution of conventional microscopy. GSDM imaging of the same sample (Fig 7B, ~20 nm resolution) clearly identifies a number of large Nef-BiFC and TGN46 structures with no apparent overlap, as well as a number of smaller structures which contain both Nef-BiFC and TGN46. SAA analysis of the GSDM image reveals that approximately 30% of Nef-BiFC does in fact associate with TGN46, with this association occurring in a region consistent with inter-protein interactions (~12 nm separation, Fig 7C and 7E), and that this degree of association is far greater than that predicted by random chance alone. This indicates that this is a *bona fide* interaction between Nef-BiFC and the Golgi, although the extent of this interaction is far smaller than previously predicted [71] or indicated by conventional microscopy (Fig 7A). In marked contrast, TGN46 shows a much smaller degree of overlap with dimerized Nef (Fig 7D); indeed, there is less TGN46 interacting with Nef-BiFC than would be predicted by random chance alone, indicating a net exclusion of Nef-BiFC from TGN46-containing vesicles (Fig 7E). How can this apparent disagreement in the molecular association of Nef-BiFC and TGN46 be explained?

Ripley’s H function analysis provides the first clue (Fig 7F), displaying a curvature typical of a heterogeneous distribution of molecules which may contain a mixture of co-clustered and unclustered sub-populations of Nef-BiFC and TGN46. OPTICS segmentation of the Nef-BiFC image, followed by quantification of the quantity of TGN46 in each Nef-BiFC cluster, revealed significant co-clustering of TGN46 and Nef-BiFC in small vesicles corresponding to the predicted size of Golgi-derived intracellular transport vesicles (20–60 nm, [73]), while larger Nef-BiFC containing vesicles were negative for TGN46 (Fig 7G). This clustering data resolved the apparent disagreement in Nef-TGN46 interactions identified by SAA analysis–the small vesicles containing co-clustered Nef-BiFC and TGN46 contained ~30% of the total Nef-BiFC and ~2% of the total TGN46 (Fig 7H), indicating that these small vesicles represented the primary site of Nef-TGN46 interactions. While these vesicles incorporated a large portion of the total cellular Nef-BiFC, they contained only a minute portion of the total cellular TGN46. Interestingly, not only did the larger Nef-BiFC containing vesicles completely exclude TGN46, but they were also closely apposed to the TGN–sufficiently closely opposed as to overlap with the TGN in conventional microscopy images (Fig 7A and 7B), thus potentially representing a previously undescribed Nef-targeted cellular niche. Thus, by applying these analytical tools to super resolution images we have identified, for the first time, a Golgi-proximal cellular niche in which dimerized Nef is sequestered.

Clearly, application of multiple analytical methods in MIiSR provides insight into intermolecular interactions, and the location of these interactions within the cellular environment, that are not detectable by other biochemical and imaging techniques. Applied to a single image, MIiSR can extract a wealth of data regarding intermolecular interactions and the formation of higher-order molecular complexes within a cell, providing a molecular view of biological systems not available through conventional imaging or biochemical methodologies.

### Availability and Future Directions

MIiSR provides a comprehensive set of analytical tools for analyzing intermolecular interactions between two or three molecular species (Spatial Association Analysis), quantification of molecular clustering and co-clustering (Ripley’s H-function, Radial Distribution Function and RDF quantification), and for the identification of individual molecular clusters (H-function segmentation, DBSCAN and OPTICS analyses) in super-resolution images. MIiSR is an open-source project licensed under the GNU General Public License and is available as S1 –MIiSR Program in this paper, with updated versions published at http://www.phagocytes.ca/miisr/. MIiSR is provided as Matlab-formatted functions and GUI’s, and has been confirmed to run properly with Matlab versions 2010b through 2015a. MIiSR works on any Linux, Windows and Macintosh computer running an appropriate version of Matlab equipped with the image processing, statistics and parallel processing toolboxes. Users may extend the functionality of MIiSR through incorporating MIiSR functions into their own Matlab functions and scripts, and through modification of the MIiSR source code. Our areas for future development include extending the range of molecular precision file formats natively supported by MIiSR and improving automated feature detection during OPTICS-based segmentation.

## Materials and Methods

### Materials

RPMI, HBSS, DMEM, EMEM, Trypsin-EDTA and Fetal Bovine Serum (FBS) were purchased from Wisent (Saint-Jean-Baptiste, Canada). Cy3 and Cy5 labeled DNA oligomers were ordered from IDT (Coralville, Iowa). #1.5 thickness round cover slips and 16% paraformaldehyde (PFA) were purchased from Electron Microscopy Supplies (Hatfield, Pennsylvania). HEK 293 and HeLa cells were purchased from ATCC (Manassas, Virginia), SN56 cells were a kind gift from Jane Rylett (University of Western Ontario, Canada). Lipofectamine 2000, Alexa Fluor-labeled secondary Fab antibodies and anti-Golgin97, were purchased form Life Technologies (Burlington, Canada). Dylight-labeled secondary Fab antibodies were purchased from Cedarlane (Burlington, Canada). Cysteamine, mouse anti-HA antibody, rabbit anti-FLAG antibody, Isoproterenol, dibutyrl cyclic AMP, Bovine Serum Albumin (BSA), anti-AP-1γ, and anti-TGN46 were purchased from Sigma-Aldrich (Oakville, Canada). Rat anti-LAMP1 antibody was purchased from the Developmental Studies Hybridoma Bank (Iowa City, Iowa). PolyJet was purchased from Frogga Bio (North York, Canada). All other chemicals were purchased from ThermoFisher (Toronto, Canada). Matlab software was purchased from MathWorks (Natick, Massachusetts). Prism software was purchased from Graphad (La Jolla, California).

### MIiSR Functions & GUI

All functions and GUI’s provided as part of the MIiSR software package were written in Matlab version R2014a and require the Parallel Processing, Image Processing, Statistics and Optimization toolboxes. DBSCAN.m was prepared by modifying previously published scripts by Daszykowsi *et al*. [59], to add the required functionalities for this toolbox and to enhance computational speed. All other scripts were written *de novo*.

### Cell Culture & Stimulation

Human embryonic kidney (HEK 293) cells were maintained in EMEM + 10% fetal bovine serum (FBS), CD4+ HeLa cells were maintained in RPMI + 10% FBS, and SN56 cells were maintained in DMEM + 10% FBS. All cells were maintained by growing to 80% confluency in a 37°C/5% CO_2_ incubator, at which point they were split 1:10 into a new flask by briefly washing the cells with pre-warmed phosphate-buffered saline (PBS, 0.9% NaCl, 10mM Na_2_HPO_4_, 2 mM KH_2_PO_4_, pH 7.4) followed by a short incubation in trypsin-EDTA. For imaging, #1.5 18mm diameter circular coverslips were first acid washed by rinsing in 100% ethanol, cleaned for ~12 hours in 1M HCl at 50–60°C with intermittent agitation, then rinsed with 100% ethanol and placed between sheets of filter paper to dry. Dried cover slips were autoclaved, placed into the wells of a 12-well tissue culture plate, and cells seeded in 1ml of media as indicated below.

HEK cells were seeded in 10 cm dishes at 70–80% density 24 h prior to transfection, and transfected with 2 μg β_2_AR-Flag, 3 μg of Clathrin-HA and 2 μg of β-arestin1-GFP as described previously [55]. Eighteen hours post-transfection the cells were washed with PBS and re-seeded at 30% confluency onto sterile acid-washed cover slips. 48 hours following transfection the cells were stimulated with 10 μM Isoproterenol for 1 or 5 minutes and then fixed and stained as described below. SN56 and CD4+ HeLa cells were plated directly onto sterile acid-washed coverslips at 30–50% confluency and transfected 12–18 hours later. SN56 cells were transfected with 2 μg of APP-GFP [60] using lipofectamine 2000 as per manufacturer’s instructions and then differentiated for 24 hours in DMEM + 10% FCS and 1 mM dibutyrl cyclic AMP prior to fixation. CD4+ HeLa cells were transfected using GenJet and 1 μg GFP-Nef and either 1 μg mCherry-PACS-1 or 1 μg mCherry-AP1 [13,44], and fixed 48 hours later unless otherwise indicated. Alternatively, CD4+ HeLa cells were transfected with 1 μg each of the Nef BiFC reporters Nef-Yn and Nef-Yc [13] and incubated 24 hours prior to fixation.

### Fixation and Immunostaining

Cells were washed once with PBS and then fixed for 20 min using 4% PFA in PBS at 20°C. Cells were blocked and permeabilized with PBS + 5% BSA and 0.1% Triton X-100 for 30 to 60 minutes. Permeabilization was employed even if fluorescent transgenes had been expressed or cell-surface staining was performed, in order to ensure access of the GSD imaging media to all fluorophores in the sample. Primary antibodies (1:200 anti-LAMP1, 1:5,000 anti-HA, 1:5,000 anti-FLAG, 1:200 anti-AP-1γ, 1:200 anti-TGN46) were then added in PBS + 5% BSA and incubated for 20 minutes (extracellular antigens) or 90 minutes (intracellular antigens). Samples were washed 3 x 15 min in PBS and dylight-488, Cy3 or dylight-647 conjugated secondary Fab’s added at a 1:1000 dilution in PBS + 5% BSA for 1 hour. Samples were washed 3 x 15 min with PBS, subjected to a 15 min secondary fixation using 2% PFA in PBS, washed 1 x 5 min in PBS, and then stored at 4°C until imaged. Immediately before imaging the samples were mounted in freshly prepared imaging media (PBS plus 100 mM cysteamine), which is required to protect the triplet (dark) state fluorophores from oxidation (photobleaching) during GSDM imaging [29]. The coverslips were then mounted on a 75 μl depression slide containing filled with imaging media and the coverslip-covered depression sealed using either the water-compatible rapid-curing silicone sealant Twinsil, or heat-set VALAP (1:1:1 by weight mixture of Vaseline (petroleum jelly), Lanolin and Paraffin). Samples were imaged within 4 hours of mounting.

### Ground State Depletion Microscopy (GSDM)

All super-resolution images were acquired using GSDM. Mounted samples were placed on the stage of a Leica SR GSD microscope equipped with a TIRF-compatible 100×/1.43 NA objective lens plus an addition 1.6× optical magnifier for a total of 160× magnification, 125 mw–250 mW imaging lasers (488, 555 and 647 nm) and a 30 mW backpumping laser (405 nm). Individual color channels were imaged sequentially starting with the longest wavelength dyes. For each channel the sample was subjected to a depletion period in which the sample was excited at maximum laser intensity until less than 120 active fluorophores were present in each image. Excitation intensity was then reduced to ~20% and the sample imaged at 100 fps for 5,000–30,000 frames with backpumping laser intensity increased over the imaging period in order to maintain 50–120 active fluorophores per frame. The resulting molecule position files were exported as either TIFFs with 20 μm pixels or as ASCII-formatted molecule position files for subsequent analysis.

### Monte Carlo Modeling of Unclustered Molecules

For many analyses the degree of colocalization or clustering needs to be compared to the degree of colocalization or clustering of an equivalent number of molecules, distributed over the same area, but in which molecules are non-interacting. Computationally-derived samples mimicking this situation were produced by randomizing molecule positions from GSDM images. First, the area of the image is determined by extracting the minimum and maximum *x*, *y* and *z* coordinates from the molecule position files. Next, the *x*, *y* and *z* coordinates of the original molecules are replaced with random coordinates, generated using a uniformly distributed random number generator, falling within the bounds of the original image. This produces images of non-interacting (e.g. randomly distributed) molecules that contain the same number of labeled molecules distributed over the same area, as the original image. These randomized images were then analyzed using the relevant MIiSR tool.

### Creation of Monomeric Fluorophore Fields

To test the SAA analysis fields of molecules with defined degrees of interactions were required. Briefly, several complementary and non-complementary DNA oligomers of varying lengths, designed to eliminate any secondary structure [74] were purchased with either 5’ Cy3 or 5’ Cy5 labels (Table 1). The Cy3 labeled DNA oligomer was then mixed with varying ratios of either the complementary or non-complementary Cy5 labeled oligo at 50 nM concentration and annealed in a thermocyler by heating to 95°C for 30 sec followed by cooling to 4°C for 5 min. The oligos were then diluted 1:100,000 in distilled water and absorbed onto an acid-cleaned cover slip for 30 min at room temperature. When a 1:1 ratio of Cy3-to-Cy5 fluorophores was used this produced a uniform field of fluorophores, with fluorophores in the same channel separated by ~60 nm (Fig 1A). To confirm monomeric distributions were being generated, these oligos diluted an additional 1:10,000 were coated onto coverslips, generating uniform fields of fluorophores which were individually resolvable by conventional microscopy. Intensity profiling and step-wise photobleaching assays of these cover slips demonstrated that the vast majority (>98%) of fluorophores were monodispersed. In subsequent GSDM imaging the Cy3 and Cy5 fluorophores were respectively detected 5.7 ± 2.1 and 3.2 ± 1.8 times, with 87.3% ± 6.9% of the Cy3 and 91.1 ± 12.7% of the Cy5 fluorophores detected in the pre-GSDM imaged sample recovered during GSDM imaging.

**Table 1:** Fluorescently labeled DNA oligomers.

Name	Sequence	5’ Tag	Length*
20-mer	CTTTTAAAGATATGATTAGA	Cy3	6.8 nm
20-mer complementary	TCTAATCATATCTTTAAAAG	Cy5	6.8 nm
30-mer	CTTTTAAAGATATGATTAGACTTCGTTAGT	Cy3	10.2 nm
30-mer complementary	ACTAACGAAGTCTAATCATATCTTTAAAAG	Cy5	10.2 nm
30-mer non-complementary	TGATTGCTTCAGATTAGTATAGAAATTTTC	Cy5	10.2 nm
40-mer	CTTTTAAAGATATGATTAGACTTCGTTAGTTGATTGCTTC	Cy3	12.8 nm
40-mer complementary	GAAGCAATCAACTAACGAAGTCTAATCATATCTTTAAAAG	Cy5	12.8 nm
50-mer	CTTTTAAAGATATGATTAGACTTCGTTAGTTGATTGCTTCAGATTAGTAT	Cy3	17.0 nm
50-mer complementary	ATACTAATCTGAAGCAATCAACTAACGAAGTCTAATCATATCTTTAAAAG	Cy5	17.0 nm
60-mer	CTTTTAAAGATATGATTAGACTTCGTTAGTTGATTGCTTCAGATTAGTATAGAAATTTTC	Cy3	20.4 nm
60-mer complementary	GAAAATTTCTATACTAATCTGAAGCAATCAACTAACGAAGTCTAATCATATCTTTAAAAG	Cy5	20.4 nm

*Length indicates the estimated length of the annealed oligo, based on the dimensions of B-DNA [43].

### SAA Analysis

For DNA oligo analysis, differing ratios of complementary and non-complementary DNA oligos were GSDM imaged as described above, or GSDM imaging was performed on Golgin-97 immunostained CD4+ HeLa cells ectopically expressing Nef-GFP and mCherry-PACS-1. SAA was conducted on the resulting 2-color (DNA oligo) and 3-color (Nef/PACS-1/Golgin97) images using SAA2col.m and SAA3col.m respectively. To model undersampling, individual Cy3 and Cy5 molecules were deleted from the DNA oligo data sets by randomly deleting, using a uniform random number generator, individual Cy3 or Cy5 molecules in a particle position file. To ensure any repeat detections of these fluorophores were also eliminated, all detected molecules falling within the same color channel and within a radius equal to the CDC (S1 Text), of these molecules were also removed from the dataset. To model oversampling, individual Cy3 and Cy5 molecules were randomly selected, using a uniform random number generator, the particles duplicated in the dataset after computationally resampling their position within the diameter of the CDC (21.27 nm). Both the oversampled and undersampled data sets were then analyzed using SAA2col.m. The full calculations for both the CDC and SAA can be found in the S1 Text.

### RDF and Ripley’s Analysis

GSDM imaging of stained HEK cells was performed as described above. The resulting molecules’ position files were cropped to the cell boundaries and converted into 3-color 16-bit TIFF images with 20 nm pixels using LoadCrop.m, provided in this toolbox. Cross-RDF and Cross-Ripley’s analysis was then performed using CrossRDF.m and CrossRipley’s.m respectively, in both cases limiting analysis to radii of 1000 nm. Oversampling and undersampling were performed as described in “SAA Analysis”, above, with the added step of converting the resulting position files into TIFF images using LoadCrop.m. A full derivation of the RDF and Ripley’s algorithms can be found in the S1 Text.

### Modeling—Image Acquisition Modeling

Modeling of changes in image autocorrelation and Ripley’s H-function during image acquisition was performed by first generating synthetic images 5,000 x 5,000 nm in size, containing 200 clusters of 100 ± 10 fluorophores with radii of 75 ± 37.5 nm; to this image 10% noise (non-clustered molecules) was added. Random positioning of clusters, cluster size and positioning of molecules within each cluster, were achieved using a linear random number generator. Cluster centers were generated as a series of random x/y coordinates. The number of molecules in each cluster was then determined by subtracting a random integer between 0 and 20 from the largest allowed cluster size, and the cluster radius determined by subtracting a randomly generated number between 0–75 from the maximum cluster radius. Molecules were then randomly placed inside of the cluster by generating a random angle and radius, and using those values to position the molecule relative to the clusters’ center point. To model undersampling, this “true” image was compared to a copy wherein 10% of fluorophores were randomly deleted. This undersampled image was then “imaged” (sampled) 10,000 times, with 10–20 randomly selected fluorophores “active” in each image. The 20 nm precision of our super-resolution microscope was modeled by randomly generating a shift in the molecular position using a normally-distributed random number generator with a full-width-at-half maximum of 0.5 the precision of the microscope (10 nm). This combination of undersampling and virtual imaging fully recapitulates the over- and under-sampling typical of super-resolution acquisitions. Autocorrelation was quantified by reconstructing the super-resolution image 50 frames at a time, and comparing Pearson’s correlation of the current image versus the image reconstructed 50 frames previously. At select intervals the reconstructed image was exported and cross-Ripley’s H Function performed using the non-undersampled image as the second color channel.

### Modeling–Ripley’s and RDF Analysis

Pre-clustered molecules were added to these models as described above, except that 50 clusters of 50 nm radius, containing 100 molecules, were generated. In all models, clusters were static (non-animated) structures. For models containing one or two species of moving molecules, the starting x and y coordinates for each moving molecule was generated using a linear random number generator. Each moving molecule was then assigned to the nearest cluster and the end point positioned within the cluster using a randomly generated angle and radius, as described above. For models quantifying the impact of increasing clustering, the moving molecules where then animated over 200 frames, during which each molecule was moved linearly closer to its end-point. To model the impact of increasing portions of unclustered molecules, co-clustered molecules were generated as above, and 2% additional unclustered molecules added to each frame over 200 frames. Once generated, all frames from each video were analyzed using the spatialStatistics.m function in MIiSR.

### DBSCAN Analysis

GSDM images of APP-GFP expressing SN56 cells immunostained for LAMP1 were acquired as described above. Ideal *k* and ε values for DBSCAN analysis were identified by repeat processing of a sample image using step-wise increases in *k* (5–200 in steps of 5) and ε (100–1000 in steps of 100). The best values, determined visually, were then applied to all images acquired on the same day. A full derivation of the DBSCAN algorithm can be found in the S1 Text. For LAMP1 density analysis, the bounding area of APP clusters was identified by applying the “convhull” (convex hull) command in Matlab to the edge points of each cluster. The number of LAMP1 molecules found within each cluster were then quantified using the “inpoly” command in Matlab, using the bounding area of the APP cluster determined previously with convhull. LAMP1 density was then determined by dividing the number of LAMP1 molecules in the APP cluster by the area of the APP cluster.

### OPTICS Analysis

The same APP-GFP/LAMP1 images as used for DBSCAN analysis (above) were analyzed using OPTICS.m with a minimum cluster size set to 0.5% of total molecules in the image. APP clusters were identified by setting a static RD threshold of 34 (determined visually), with clusters defined as “valleys” bound by peaks with RD values greater than 34. LAMP1 clusters were identified by hierarchally segmenting the RD plot at peaks in the RD data using hierOPTICS.m, with minimum cluster size set to 5 molecules and split significance set to a mean segment RD ≤ 75% the RD of the peak used for the split. The resulting RD plots were generated in Matlab, and the segmentation dendograms manually added during figure preparation. A full derivation of the OPTICS algorithm and our hierarchal segmentation algorithm can be found in the S1 Text.

### Statistical Analysis

All experiments were repeated at least 3 times, with at least 5 images collected during each experiment. Statistical analyses were performed in either Matlab or Graphpad Prism. Manders ratios were calculated using ImageJ equipped with the Just Another Colocalization Plugin [18]. Statistical significance was set as p ≤ 0.05. Unless otherwise noted a Students *t*-test was used for all two-sample experiments and ANOVA with Bonferroni correction used for all multi-sample experiments.

## Supporting Information

S1 Fig Spatial association analysis–Operation and effect of over- and under-sampling. (A) Spatial association analysis functions by assessing distances between the molecule of interest (blue circles) and the nearest-neighbour molecule in the other colour channel (red circles). The intermolecular separation distances between nearest neighbours is calculated for all molecules in the primary (blue) channel, and the resulting measurements plotted as a histogram (“Measured” in histogram). The positions of the molecules in the second channel are then randomized (light-red molecules) and the measurement repeated (“Randomized” in histogram). Intermolecular interactions are indicated by an enrichment of neighbouring molecular pairs with small nearest-neighbor separation distances. (B) Effect of oversampling on the measured degree of association of annealed, complementary 30mer (~10.2 nm) DNA oligomers 5’ labeled with Cy3 and Cy5. (C) Effect of undersampling on the measured degree of association of annealed, complementary 30mer (~10.2 nm) DNA oligomers 5’ labeled with Cy3 and Cy5. (D) Effect of undersampling on the fold-increase observed between the measured numbers of interacting neighbours relative to the predicted number of interacting neighbors based on image randomization (Fold Increase). Analysis is conducted using 30-mer (~10.2 nm) DNA oligomers 5’ labeled with Cy3 and Cy5. (E) Impact of oversampling on same-channel SAA analysis of randomly dispersed 30mer DNA oligomers 5’ labeled with Cy3. Data are presented as the mean ± SEM (B-D) or is representative of, 3 independent experiments with a minimum of 3 images per experiment. n.s. = no statistical differences relative to non-oversampled or undersampled Cy3 and Cy5. * = p < 0.05 compared to non-undersampled or oversampled Cy3, † = p < 0.05 compared to non-undersampled or oversampled Cy5.
(TIF)

S2 Fig Radial distribution function–Operation and effect of over- and under-sampling. (A) RDF (pair-correlation or G(r)) analysis operates by quantifying the number of molecules (blue points) in an expanding torus centered on each molecule, normalized to the average density of molecules in the sample. For an unclustered dataset, G will have a value of 1 at all radii (Unclustered). For datasets with regularly spaced clusters, the mean cluster radius is indicated by the distance between the graph origin and the first peak in the data (*w*, top graph), with the distance between clusters indicated by the distance between subsequent peaks (*m*, top graph). Biological samples, which rarely have regularly spaced clusters, generally display only the first peak, allowing for assessment of mean cluster size (*w*, bottom graph). (B) Modeled negative effects of over-sampling in single-channel RDF, generated by computationally re-sampling images to add repeat fluorophore detections. (C) Cross-correlation (cross-RDF) analysis is performed the same as conventional RDF analysis, except the function assesses the number of particles in a second colour channel (blue particles) relative to the number of particles in the first colour channel (red particles). (D) Cross-correlation analysis is not impacted by high (25×) oversampling and is minimally impacted by undersampling. Data are shown as graphs (B, D) plotted from representative images from 3 experiments, minimum of 5 images per experiment.
(TIF)

S3 Fig Ripley’s K-function–Operation and effect of over- and under-sampling. (A) Ripley’s K function operates by quantifying the density of particles (blue points) in an expanding circular area around each point, compared to the number of points predicted given the average point density of the image. When K is plotted against radius, clustering appears as higher-than predicted K values at small radii, and lower-than expected values at larger radii (Clustered versus unclustered, top graph). The H-derivative (bottom graph) improves cluster detection by normalizing the value of K to both area and radius. Unclustered molecules will have an H value of zero for all radii, while clustered data will produced peaked data, where the average cluster radius is indicated by the peak of the graph (arrow) and the average separation distance is indicate as two times the radius at which the H(r) plot re-crosses the zero line. (B) Modeled negative effects of over-sampling in single-channel Ripley’s H-function analysis, generated by computationally re-sampling images to add repeat fluorophore detections. (C) Cross-Ripley’s analysis is performed the same as conventional Ripley’s analysis, except that the function assesses the number of particles in a second colour channel (blue particles) relative to the number of particles in the first colour channel (red particles). (D) Example of a super-resolution reconstruction of a GSDM image acquisition, using a modeled sample (Original) that has been computationally imaged. Undersampled (frames 50, 500 and 100), near-ideally sampled (frame 2500) and oversampled (frame 10,000) frames are shown. (E) The degree of autocorrelation observed during reconstruction of the image show in D raises quickly early in image reconstruction, and asymptotically approaches 1 later in the reconstruction. Autocorrelation was calculated between frames 1…n and frame 1…(n+50) of the super-resolution acquisition series. (F) The mean cluster size measured by Ripley’s H analysis most closely represents an ideally sampled (no over- or–undersampling, “Original”) image when the sampled image has a Pearson’s autocorrelation of ~0.990. Panels B, D, E and F are representative of 5 independent experiments.
(TIF)

S4 Fig Operation of the DBSCAN and OPTICS algorithms. (A) The DBSCAN algorithm requires the user to provide the minimum number of particles in a cluster (*k*) and the neighbourhood size in which a cluster would exist (ε). Assuming *k* = 5, molecule ‘*a*’ (green) would be considered *directly density reachable*, and thus a core molecule in a cluster, as more than 5 particles are found within its ε-neighborhood (green circle). Molecule ‘*b*’ (orange) would be considered *density reachable*, and thus an edge point, as it falls within the ε-neighbourhood of a core molecule (A), but does not contain 5 neighbours within its ε-neighborhood. Molecule ‘*c*’ (blue) is not density-reachable from any particle at this value of ε, and therefore would be considered non-clustered. (B) DBSCAN-mediated clustering of modeled data using varying values of *k* and ε. Because DBSCAN uses static values of ε and *k* to determine density, it has difficulty separating clusters of differing density (green) or separating noise surrounding clusters with densities > ε (red). (C) The OPTICS algorithm measures the local neighborhood density by ordering the molecules in the dataset by proximity, and then measuring two quantities for each molecule. The first is the *core-distance*, defined as the distance from the current molecule to the *k* ^th^ closest molecule, where *k* is a user-selected minimum cluster size (left panels, molecules *a* and *b*). Secondly, the distance from the current molecule to the next molecule in the list (molecules *1*–*5*, right panel) is measured. The larger of these two values is then kept as the *Reachability Distance* (RD). D) Reachability plot of the same data set shown in panel B generated using OPTICS with *k* = 15. Clusters appear as “valleys” separated by “peaks” in the RD plot. Automatic cluster detection, using a hierarchal segmentation of the RD plot, with the segmentation pattern shown as a dendogram overlaid on the RD plot. Individual clusters are identified by the colored or dotted horizontal lines. (H) Clusters identified by OPTICS, highlighting the good separation of clusters even with the presence of clusters of differing density and clusters in close proximity.
(TIF)

S1 File MIiSR program. This zip-compressed archive contains the MIiSRconvert and MIiSR GUI’s, as well as stand-alone function for scripts for converting common super-resolution microscopy position files to Matlab-compatible matrices (fileConv.m), for cropping and filtering super-resolution microscopy position files (LoadCrop.m), and to perform the SAA (SAA2col.m, SAA3col.m), RDF and Ripley’s analysis (spatialStats.m), DBSCAN (DBSCAN.m), OPTICS (OPTICS.m) and hierarchal segmentation of the OPTICS plot (hierOPTICS.m) analyses described in this paper. In addition, stand-alone functions for Owen *et al’s*. Ripley’s H-function based image segmentation (Hsegment.m, [39]) and Sengupta *et al’s*. quantification of the number molecules in an RDF plot (RDFquant.m, [15]) have been included. All of these analyses can be performed using either 2D or 3D molecule position files. The use and dependencies of each function can be found in the header of each Matlab function.
(ZIP)

S1 Text Derivation of the SAA, RDF, Ripley’s, DBSCAN and OPTICS algorithm. Complete derivation of all algorithms used in the MIiSR toolbox.
(DOCX)

S1 Video Evolution of RDF and Ripley’s H-function curves with increasing clustering of two initially unclustered populations. Model of two randomly distributed populations of 5,000 molecules/population (red and blue dots, left panel) which coalesce into 50 co-clusters. Upper-right panel display’s cross-Ripley’s H function for each frame, while the lower-right panel displays the cross-RDF function for each frame.
(MPG)

S2 Video Evolution of RDF and Ripley’s H-function curves with increasing clustering of a random population with a pre-clustered population. Model of one randomly distributed population of 5,000 molecules (blue dots) which coalesce into 50 pre-existing clusters of red molecules. Upper-right panel display’s cross-Ripley’s H function for each frame, while the lower-right panel displays the cross-RDF function for each frame.
(MPG)

S3 Video Impact of partial clustering on RDF and Ripley’s H-function. Model of two co-clustered molecules (5,000/color) with an increasing portion of unclustered molecules. Each frame 200 additional unclustered molecules are added to each population (left panel). Upper-right panel display’s cross-Ripley’s H function for each frame, while the lower-right panel displays the cross-RDF function for each frame.
(MPG)
