# Supplementary figures and images for: MIiSR: Molecular Interactions in Super-Resolution Imaging Enables the Analysis of Protein Interactions, Dynamics and Formation of Multi-protein Structures
Source: PLoS Comput Biol. 2015 Dec 11;11(12):e1004634. doi: 10.1371/journal.pcbi.1004634 (PMC4676698; doi:10.1371/journal.pcbi.1004634)

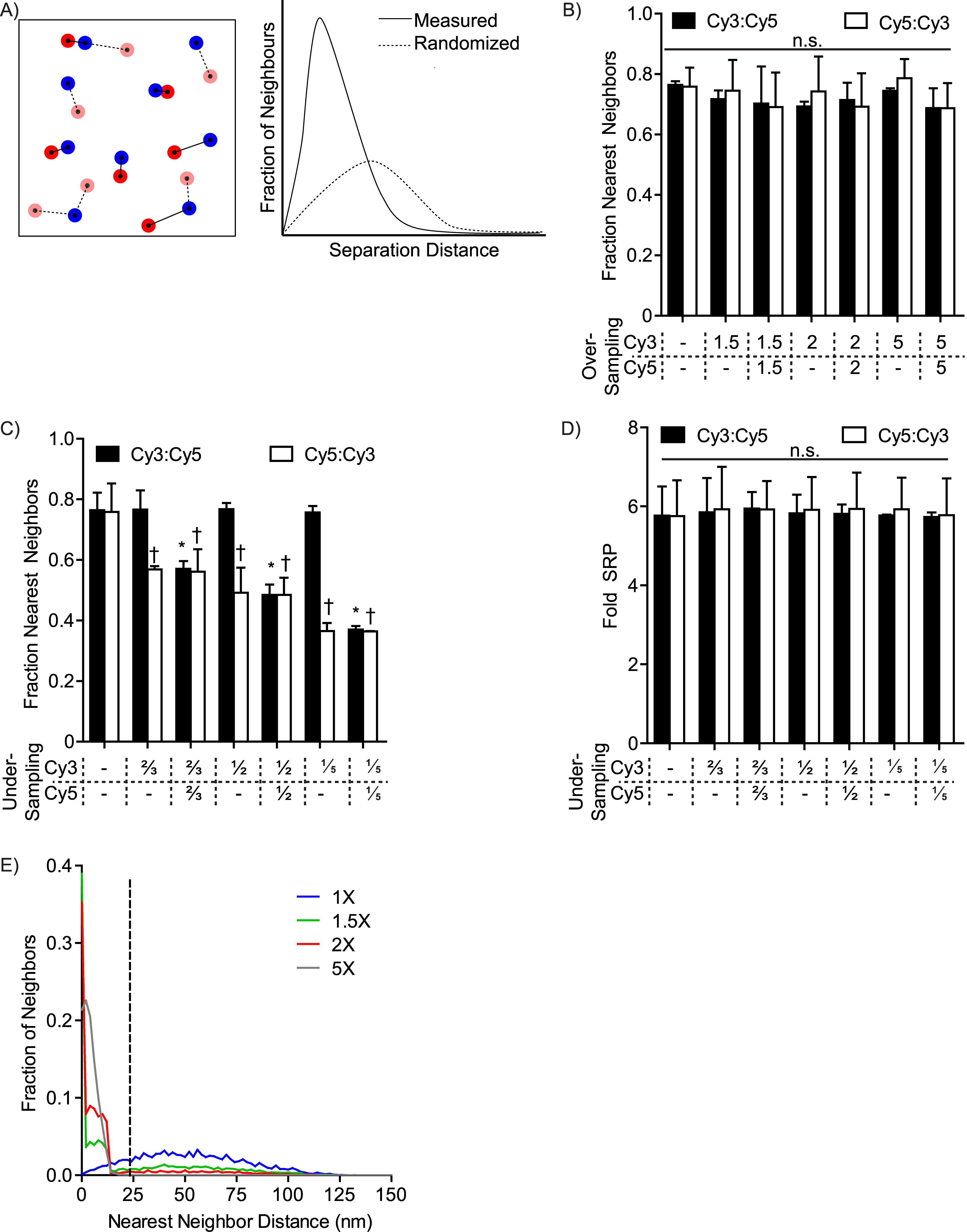

Supplement: S1 Fig — (A) Spatial association analysis functions by assessing distances between the molecule of interest (blue circles) and the nearest-neighbour molecule in the other colour channel (red circles). The intermolecular separation distances between nearest neighbours is calculated for all molecules in the primary (blue) channel, and the resulting measurements plotted as a histogram (“Measured” in histogram). The positions of the molecules in the second channel are then randomized (light-red molecules) and the measurement repeated (“Randomized” in histogram). Intermolecular interactions are indicated by an enrichment of neighbouring molecular pairs with small nearest-neighbor separation distances. (B) Effect of oversampling on the measured degree of association of annealed, complementary 30mer (~10.2 nm) DNA oligomers 5’ labeled with Cy3 and Cy5. (C) Effect of undersampling on the measured degree of association of annealed, complementary 30mer (~10.2 nm) DNA oligomers 5’ labeled with Cy3 and Cy5. (D) Effect of undersampling on the fold-increase observed between the measured numbers of interacting neighbours relative to the predicted number of interacting neighbors based on image randomization (Fold Increase). Analysis is conducted using 30-mer (~10.2 nm) DNA oligomers 5’ labeled with Cy3 and Cy5. (E) Impact of oversampling on same-channel SAA analysis of randomly dispersed 30mer DNA oligomers 5’ labeled with Cy3. Data are presented as the mean ± SEM (B-D) or is representative of, 3 independent experiments with a minimum of 3 images per experiment. n.s. = no statistical differences relative to non-oversampled or undersampled Cy3 and Cy5. * = p < 0.05 compared to non-undersampled or oversampled Cy3, † = p < 0.05 compared to non-undersampled or oversampled Cy5. (TIF) [file pcbi.1004634.s001.tif]

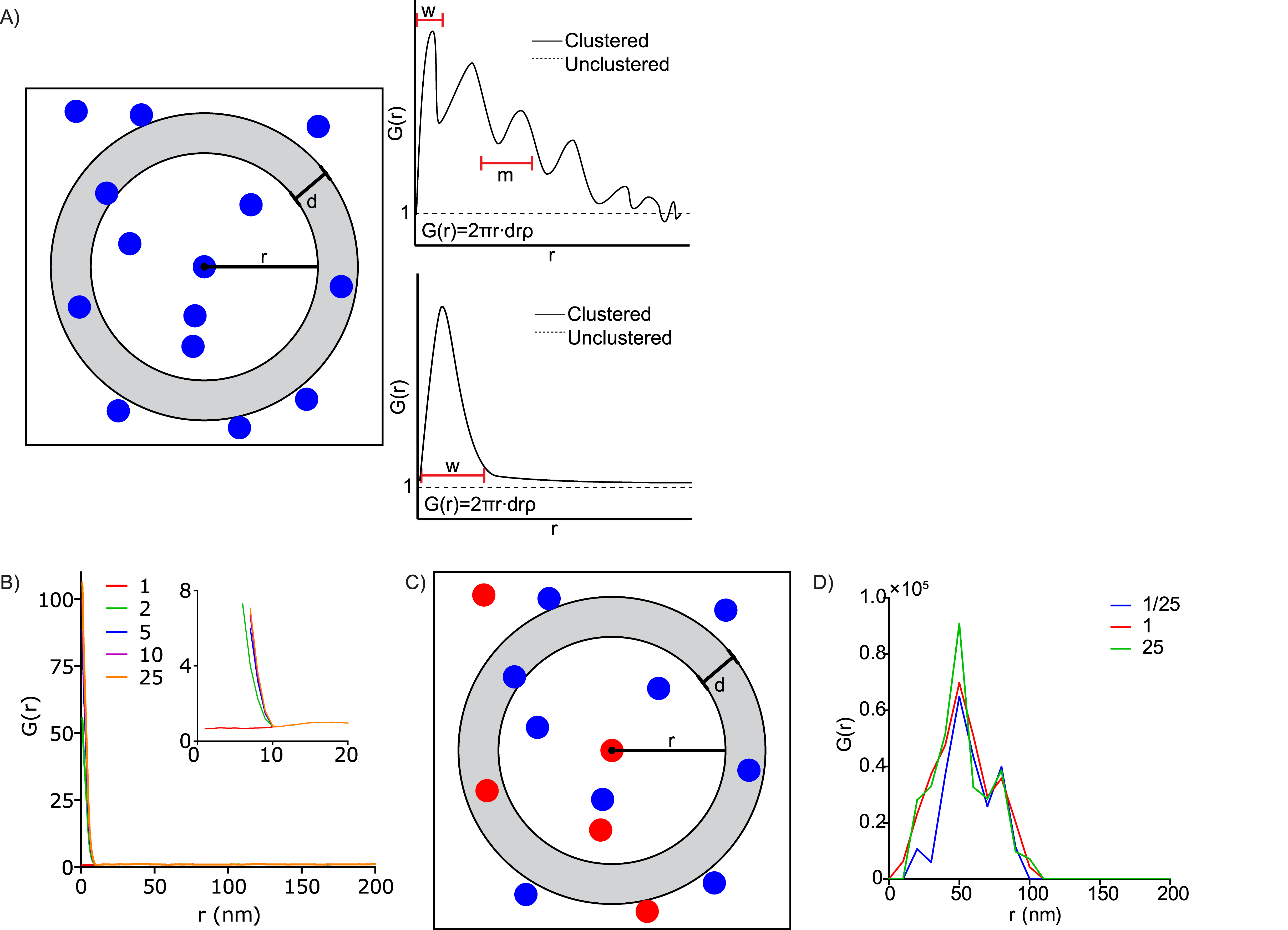

Supplement: S2 Fig — (A) RDF (pair-correlation or G(r)) analysis operates by quantifying the number of molecules (blue points) in an expanding torus centered on each molecule, normalized to the average density of molecules in the sample. For an unclustered dataset, G will have a value of 1 at all radii (Unclustered). For datasets with regularly spaced clusters, the mean cluster radius is indicated by the distance between the graph origin and the first peak in the data (w, top graph), with the distance between clusters indicated by the distance between subsequent peaks (m, top graph). Biological samples, which rarely have regularly spaced clusters, generally display only the first peak, allowing for assessment of mean cluster size (w, bottom graph). (B) Modeled negative effects of over-sampling in single-channel RDF, generated by computationally re-sampling images to add repeat fluorophore detections. (C) Cross-correlation (cross-RDF) analysis is performed the same as conventional RDF analysis, except the function assesses the number of particles in a second colour channel (blue particles) relative to the number of particles in the first colour channel (red particles). (D) Cross-correlation analysis is not impacted by high (25×) oversampling and is minimally impacted by undersampling. Data are shown as graphs (B, D) plotted from representative images from 3 experiments, minimum of 5 images per experiment. (TIF) [file pcbi.1004634.s002.tif]

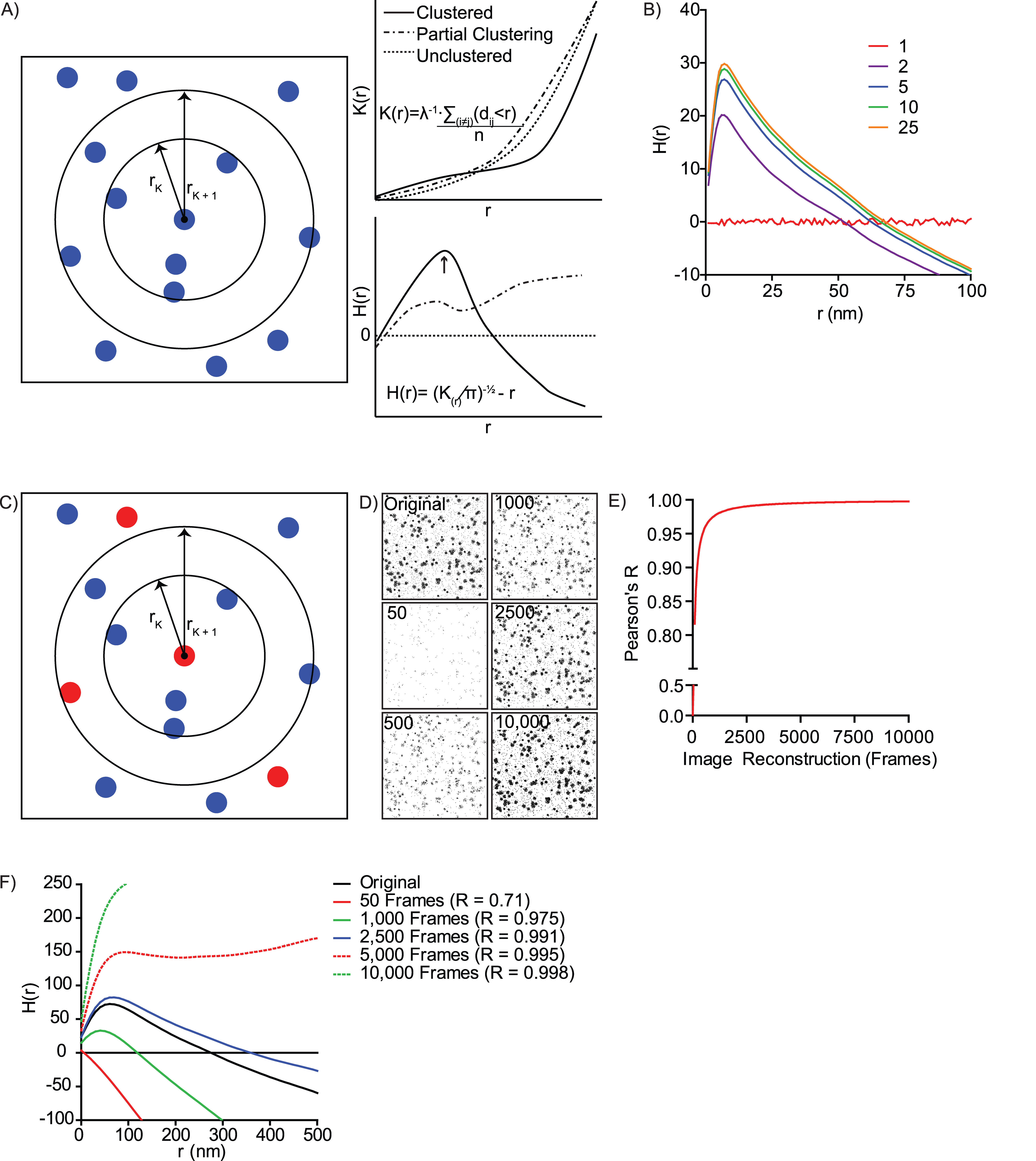

Supplement: S3 Fig — (A) Ripley’s K function operates by quantifying the density of particles (blue points) in an expanding circular area around each point, compared to the number of points predicted given the average point density of the image. When K is plotted against radius, clustering appears as higher-than predicted K values at small radii, and lower-than expected values at larger radii (Clustered versus unclustered, top graph). The H-derivative (bottom graph) improves cluster detection by normalizing the value of K to both area and radius. Unclustered molecules will have an H value of zero for all radii, while clustered data will produced peaked data, where the average cluster radius is indicated by the peak of the graph (arrow) and the average separation distance is indicate as two times the radius at which the H(r) plot re-crosses the zero line. (B) Modeled negative effects of over-sampling in single-channel Ripley’s H-function analysis, generated by computationally re-sampling images to add repeat fluorophore detections. (C) Cross-Ripley’s analysis is performed the same as conventional Ripley’s analysis, except that the function assesses the number of particles in a second colour channel (blue particles) relative to the number of particles in the first colour channel (red particles). (D) Example of a super-resolution reconstruction of a GSDM image acquisition, using a modeled sample (Original) that has been computationally imaged. Undersampled (frames 50, 500 and 100), near-ideally sampled (frame 2500) and oversampled (frame 10,000) frames are shown. (E) The degree of autocorrelation observed during reconstruction of the image show in D raises quickly early in image reconstruction, and asymptotically approaches 1 later in the reconstruction. Autocorrelation was calculated between frames 1…n and frame 1…(n+50) of the super-resolution acquisition series. (F) The mean cluster size measured by Ripley’s H analysis most closely represents an ideally sampled (no over- or–undersamplin [file pcbi.1004634.s003.tif]

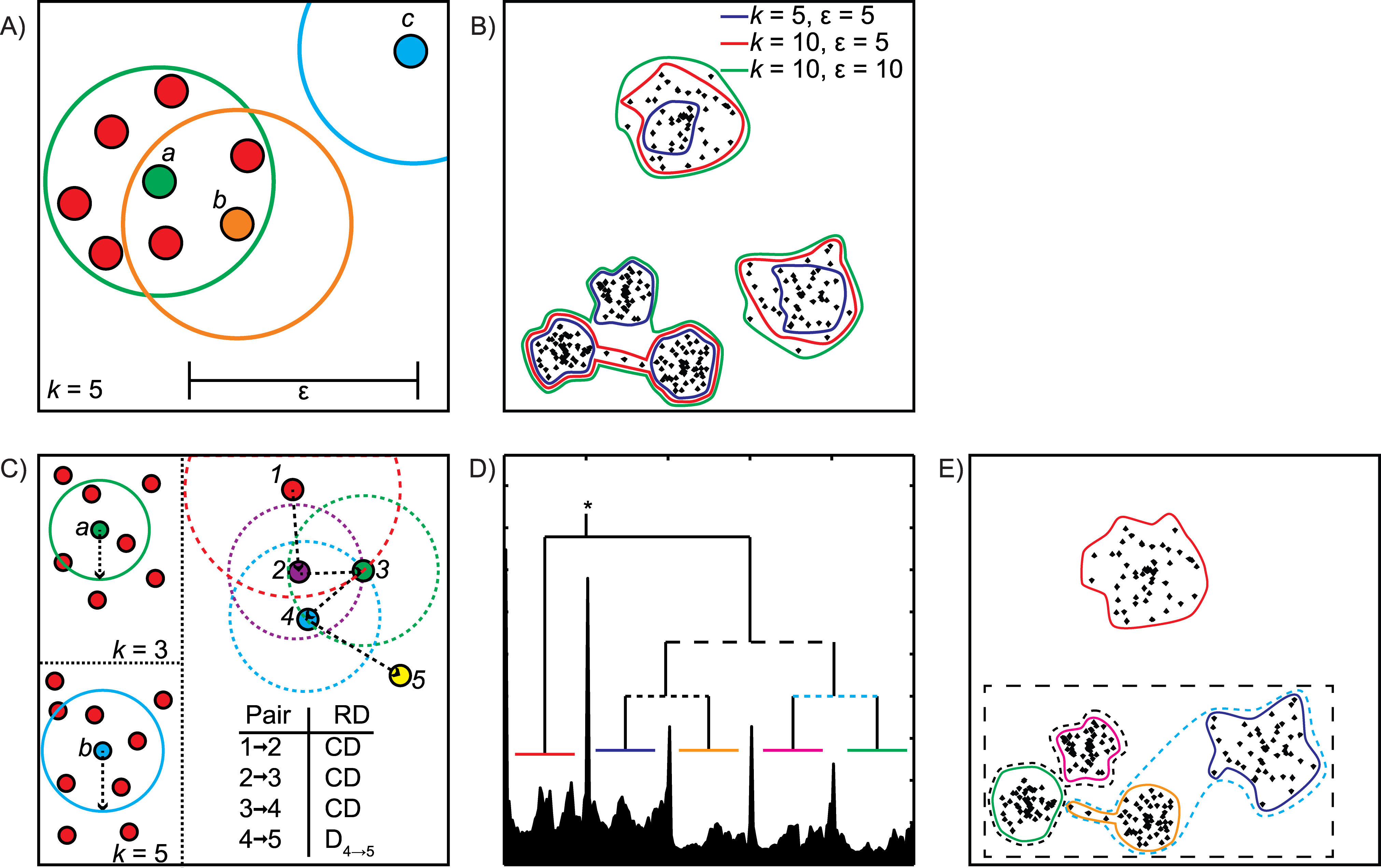

Supplement: S4 Fig — (A) The DBSCAN algorithm requires the user to provide the minimum number of particles in a cluster (k) and the neighbourhood size in which a cluster would exist (ε). Assuming k = 5, molecule ‘a’ (green) would be considered directly density reachable, and thus a core molecule in a cluster, as more than 5 particles are found within its ε-neighborhood (green circle). Molecule ‘b’ (orange) would be considered density reachable, and thus an edge point, as it falls within the ε-neighbourhood of a core molecule (A), but does not contain 5 neighbours within its ε-neighborhood. Molecule ‘c’ (blue) is not density-reachable from any particle at this value of ε, and therefore would be considered non-clustered. (B) DBSCAN-mediated clustering of modeled data using varying values of k and ε. Because DBSCAN uses static values of ε and k to determine density, it has difficulty separating clusters of differing density (green) or separating noise surrounding clusters with densities > ε (red). (C) The OPTICS algorithm measures the local neighborhood density by ordering the molecules in the dataset by proximity, and then measuring two quantities for each molecule. The first is the core-distance, defined as the distance from the current molecule to the k th closest molecule, where k is a user-selected minimum cluster size (left panels, molecules a and b). Secondly, the distance from the current molecule to the next molecule in the list (molecules 1–5, right panel) is measured. The larger of these two values is then kept as the Reachability Distance (RD). D) Reachability plot of the same data set shown in panel B generated using OPTICS with k = 15. Clusters appear as “valleys” separated by “peaks” in the RD plot. Automatic cluster detection, using a hierarchal segmentation of the RD plot, with the segmentation pattern shown as a dendogram overlaid on the RD plot. Individual clusters are identified by the colored or dotted horizontal lines. (H) Clusters identified by OPTICS, highlighting t [file pcbi.1004634.s004.tif]

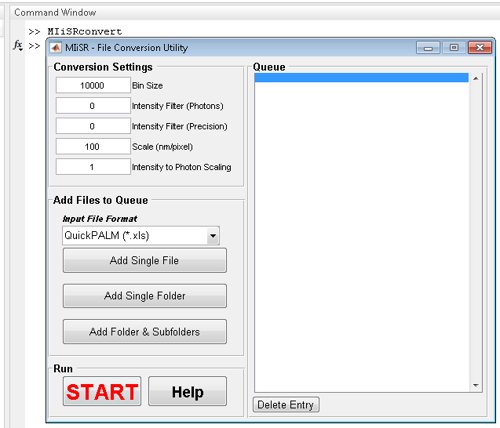

Supplement: S1 File — This zip-compressed archive contains the MIiSRconvert and MIiSR GUI’s, as well as stand-alone function for scripts for converting common super-resolution microscopy position files to Matlab-compatible matrices (fileConv.m), for cropping and filtering super-resolution microscopy position files (LoadCrop.m), and to perform the SAA (SAA2col.m, SAA3col.m), RDF and Ripley’s analysis (spatialStats.m), DBSCAN (DBSCAN.m), OPTICS (OPTICS.m) and hierarchal segmentation of the OPTICS plot (hierOPTICS.m) analyses described in this paper. In addition, stand-alone functions for Owen et al’s. Ripley’s H-function based image segmentation (Hsegment.m, [39]) and Sengupta et al’s. quantification of the number molecules in an RDF plot (RDFquant.m, [15]) have been included. All of these analyses can be performed using either 2D or 3D molecule position files. The use and dependencies of each function can be found in the header of each Matlab function. (ZIP) [file pcbi.1004634.s005.zip › help/conv01.jpg]

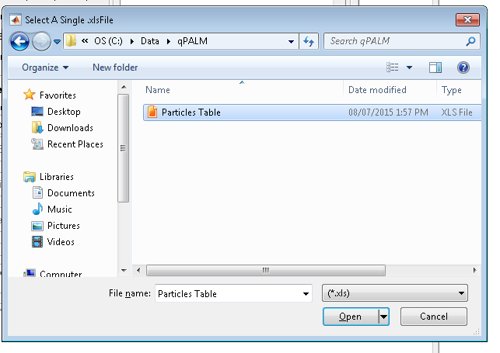

Supplement: S1 File — This zip-compressed archive contains the MIiSRconvert and MIiSR GUI’s, as well as stand-alone function for scripts for converting common super-resolution microscopy position files to Matlab-compatible matrices (fileConv.m), for cropping and filtering super-resolution microscopy position files (LoadCrop.m), and to perform the SAA (SAA2col.m, SAA3col.m), RDF and Ripley’s analysis (spatialStats.m), DBSCAN (DBSCAN.m), OPTICS (OPTICS.m) and hierarchal segmentation of the OPTICS plot (hierOPTICS.m) analyses described in this paper. In addition, stand-alone functions for Owen et al’s. Ripley’s H-function based image segmentation (Hsegment.m, [39]) and Sengupta et al’s. quantification of the number molecules in an RDF plot (RDFquant.m, [15]) have been included. All of these analyses can be performed using either 2D or 3D molecule position files. The use and dependencies of each function can be found in the header of each Matlab function. (ZIP) [file pcbi.1004634.s005.zip › help/conv02.jpg]

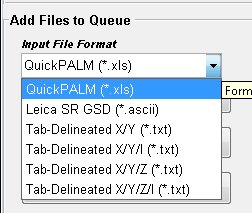

Supplement: S1 File — This zip-compressed archive contains the MIiSRconvert and MIiSR GUI’s, as well as stand-alone function for scripts for converting common super-resolution microscopy position files to Matlab-compatible matrices (fileConv.m), for cropping and filtering super-resolution microscopy position files (LoadCrop.m), and to perform the SAA (SAA2col.m, SAA3col.m), RDF and Ripley’s analysis (spatialStats.m), DBSCAN (DBSCAN.m), OPTICS (OPTICS.m) and hierarchal segmentation of the OPTICS plot (hierOPTICS.m) analyses described in this paper. In addition, stand-alone functions for Owen et al’s. Ripley’s H-function based image segmentation (Hsegment.m, [39]) and Sengupta et al’s. quantification of the number molecules in an RDF plot (RDFquant.m, [15]) have been included. All of these analyses can be performed using either 2D or 3D molecule position files. The use and dependencies of each function can be found in the header of each Matlab function. (ZIP) [file pcbi.1004634.s005.zip › help/conv03.jpg]

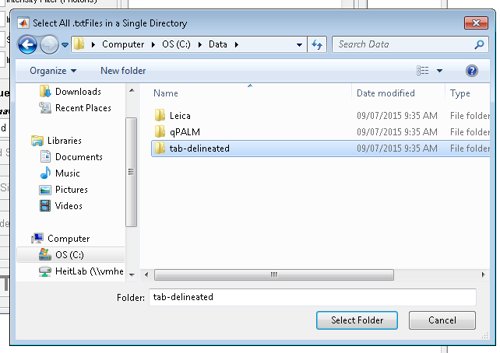

Supplement: S1 File — This zip-compressed archive contains the MIiSRconvert and MIiSR GUI’s, as well as stand-alone function for scripts for converting common super-resolution microscopy position files to Matlab-compatible matrices (fileConv.m), for cropping and filtering super-resolution microscopy position files (LoadCrop.m), and to perform the SAA (SAA2col.m, SAA3col.m), RDF and Ripley’s analysis (spatialStats.m), DBSCAN (DBSCAN.m), OPTICS (OPTICS.m) and hierarchal segmentation of the OPTICS plot (hierOPTICS.m) analyses described in this paper. In addition, stand-alone functions for Owen et al’s. Ripley’s H-function based image segmentation (Hsegment.m, [39]) and Sengupta et al’s. quantification of the number molecules in an RDF plot (RDFquant.m, [15]) have been included. All of these analyses can be performed using either 2D or 3D molecule position files. The use and dependencies of each function can be found in the header of each Matlab function. (ZIP) [file pcbi.1004634.s005.zip › help/conv04.jpg]

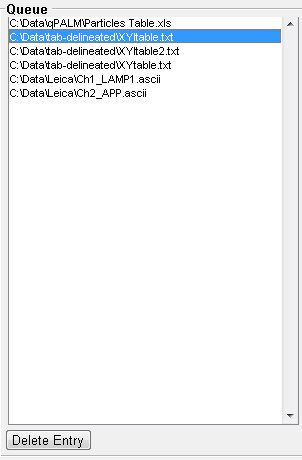

Supplement: S1 File — This zip-compressed archive contains the MIiSRconvert and MIiSR GUI’s, as well as stand-alone function for scripts for converting common super-resolution microscopy position files to Matlab-compatible matrices (fileConv.m), for cropping and filtering super-resolution microscopy position files (LoadCrop.m), and to perform the SAA (SAA2col.m, SAA3col.m), RDF and Ripley’s analysis (spatialStats.m), DBSCAN (DBSCAN.m), OPTICS (OPTICS.m) and hierarchal segmentation of the OPTICS plot (hierOPTICS.m) analyses described in this paper. In addition, stand-alone functions for Owen et al’s. Ripley’s H-function based image segmentation (Hsegment.m, [39]) and Sengupta et al’s. quantification of the number molecules in an RDF plot (RDFquant.m, [15]) have been included. All of these analyses can be performed using either 2D or 3D molecule position files. The use and dependencies of each function can be found in the header of each Matlab function. (ZIP) [file pcbi.1004634.s005.zip › help/conv05.jpg]

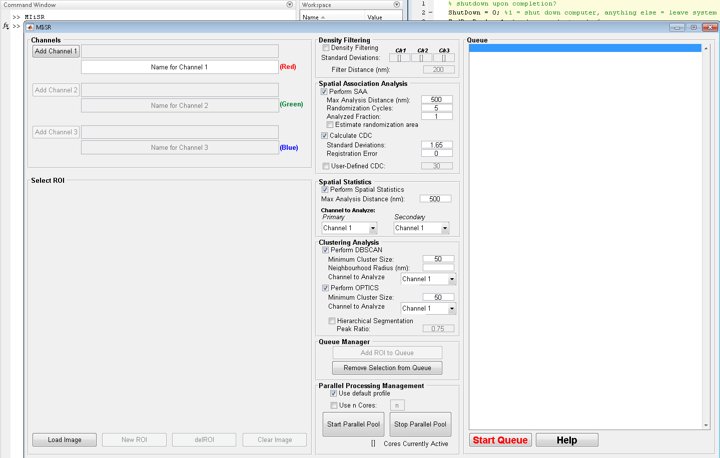

Supplement: S1 File — This zip-compressed archive contains the MIiSRconvert and MIiSR GUI’s, as well as stand-alone function for scripts for converting common super-resolution microscopy position files to Matlab-compatible matrices (fileConv.m), for cropping and filtering super-resolution microscopy position files (LoadCrop.m), and to perform the SAA (SAA2col.m, SAA3col.m), RDF and Ripley’s analysis (spatialStats.m), DBSCAN (DBSCAN.m), OPTICS (OPTICS.m) and hierarchal segmentation of the OPTICS plot (hierOPTICS.m) analyses described in this paper. In addition, stand-alone functions for Owen et al’s. Ripley’s H-function based image segmentation (Hsegment.m, [39]) and Sengupta et al’s. quantification of the number molecules in an RDF plot (RDFquant.m, [15]) have been included. All of these analyses can be performed using either 2D or 3D molecule position files. The use and dependencies of each function can be found in the header of each Matlab function. (ZIP) [file pcbi.1004634.s005.zip › help/MIiSR01.jpg]

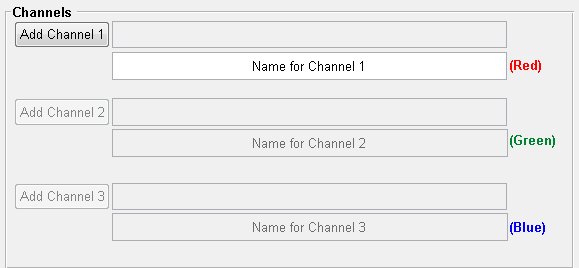

Supplement: S1 File — This zip-compressed archive contains the MIiSRconvert and MIiSR GUI’s, as well as stand-alone function for scripts for converting common super-resolution microscopy position files to Matlab-compatible matrices (fileConv.m), for cropping and filtering super-resolution microscopy position files (LoadCrop.m), and to perform the SAA (SAA2col.m, SAA3col.m), RDF and Ripley’s analysis (spatialStats.m), DBSCAN (DBSCAN.m), OPTICS (OPTICS.m) and hierarchal segmentation of the OPTICS plot (hierOPTICS.m) analyses described in this paper. In addition, stand-alone functions for Owen et al’s. Ripley’s H-function based image segmentation (Hsegment.m, [39]) and Sengupta et al’s. quantification of the number molecules in an RDF plot (RDFquant.m, [15]) have been included. All of these analyses can be performed using either 2D or 3D molecule position files. The use and dependencies of each function can be found in the header of each Matlab function. (ZIP) [file pcbi.1004634.s005.zip › help/MIiSR02.jpg]

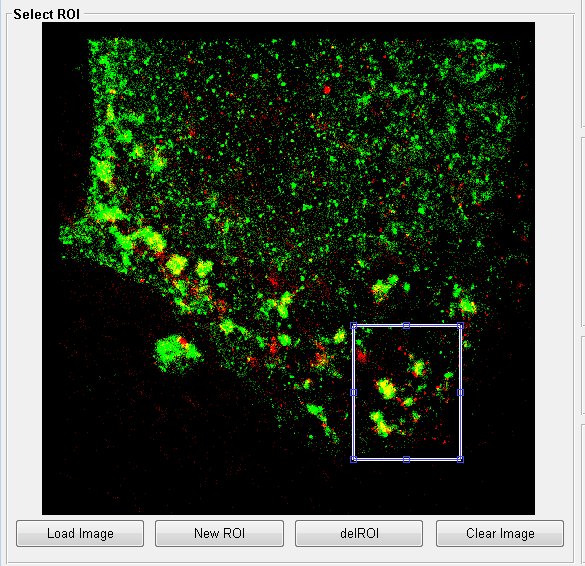

Supplement: S1 File — This zip-compressed archive contains the MIiSRconvert and MIiSR GUI’s, as well as stand-alone function for scripts for converting common super-resolution microscopy position files to Matlab-compatible matrices (fileConv.m), for cropping and filtering super-resolution microscopy position files (LoadCrop.m), and to perform the SAA (SAA2col.m, SAA3col.m), RDF and Ripley’s analysis (spatialStats.m), DBSCAN (DBSCAN.m), OPTICS (OPTICS.m) and hierarchal segmentation of the OPTICS plot (hierOPTICS.m) analyses described in this paper. In addition, stand-alone functions for Owen et al’s. Ripley’s H-function based image segmentation (Hsegment.m, [39]) and Sengupta et al’s. quantification of the number molecules in an RDF plot (RDFquant.m, [15]) have been included. All of these analyses can be performed using either 2D or 3D molecule position files. The use and dependencies of each function can be found in the header of each Matlab function. (ZIP) [file pcbi.1004634.s005.zip › help/MIiSR03.jpg]

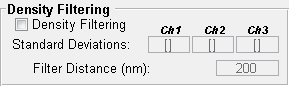

Supplement: S1 File — This zip-compressed archive contains the MIiSRconvert and MIiSR GUI’s, as well as stand-alone function for scripts for converting common super-resolution microscopy position files to Matlab-compatible matrices (fileConv.m), for cropping and filtering super-resolution microscopy position files (LoadCrop.m), and to perform the SAA (SAA2col.m, SAA3col.m), RDF and Ripley’s analysis (spatialStats.m), DBSCAN (DBSCAN.m), OPTICS (OPTICS.m) and hierarchal segmentation of the OPTICS plot (hierOPTICS.m) analyses described in this paper. In addition, stand-alone functions for Owen et al’s. Ripley’s H-function based image segmentation (Hsegment.m, [39]) and Sengupta et al’s. quantification of the number molecules in an RDF plot (RDFquant.m, [15]) have been included. All of these analyses can be performed using either 2D or 3D molecule position files. The use and dependencies of each function can be found in the header of each Matlab function. (ZIP) [file pcbi.1004634.s005.zip › help/MIiSR04.jpg]

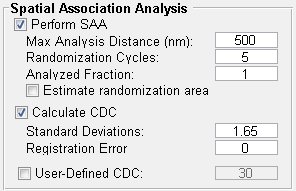

Supplement: S1 File — This zip-compressed archive contains the MIiSRconvert and MIiSR GUI’s, as well as stand-alone function for scripts for converting common super-resolution microscopy position files to Matlab-compatible matrices (fileConv.m), for cropping and filtering super-resolution microscopy position files (LoadCrop.m), and to perform the SAA (SAA2col.m, SAA3col.m), RDF and Ripley’s analysis (spatialStats.m), DBSCAN (DBSCAN.m), OPTICS (OPTICS.m) and hierarchal segmentation of the OPTICS plot (hierOPTICS.m) analyses described in this paper. In addition, stand-alone functions for Owen et al’s. Ripley’s H-function based image segmentation (Hsegment.m, [39]) and Sengupta et al’s. quantification of the number molecules in an RDF plot (RDFquant.m, [15]) have been included. All of these analyses can be performed using either 2D or 3D molecule position files. The use and dependencies of each function can be found in the header of each Matlab function. (ZIP) [file pcbi.1004634.s005.zip › help/MIiSR05.jpg]

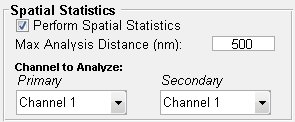

Supplement: S1 File — This zip-compressed archive contains the MIiSRconvert and MIiSR GUI’s, as well as stand-alone function for scripts for converting common super-resolution microscopy position files to Matlab-compatible matrices (fileConv.m), for cropping and filtering super-resolution microscopy position files (LoadCrop.m), and to perform the SAA (SAA2col.m, SAA3col.m), RDF and Ripley’s analysis (spatialStats.m), DBSCAN (DBSCAN.m), OPTICS (OPTICS.m) and hierarchal segmentation of the OPTICS plot (hierOPTICS.m) analyses described in this paper. In addition, stand-alone functions for Owen et al’s. Ripley’s H-function based image segmentation (Hsegment.m, [39]) and Sengupta et al’s. quantification of the number molecules in an RDF plot (RDFquant.m, [15]) have been included. All of these analyses can be performed using either 2D or 3D molecule position files. The use and dependencies of each function can be found in the header of each Matlab function. (ZIP) [file pcbi.1004634.s005.zip › help/MIiSR06.jpg]

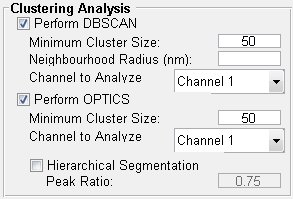

Supplement: S1 File — This zip-compressed archive contains the MIiSRconvert and MIiSR GUI’s, as well as stand-alone function for scripts for converting common super-resolution microscopy position files to Matlab-compatible matrices (fileConv.m), for cropping and filtering super-resolution microscopy position files (LoadCrop.m), and to perform the SAA (SAA2col.m, SAA3col.m), RDF and Ripley’s analysis (spatialStats.m), DBSCAN (DBSCAN.m), OPTICS (OPTICS.m) and hierarchal segmentation of the OPTICS plot (hierOPTICS.m) analyses described in this paper. In addition, stand-alone functions for Owen et al’s. Ripley’s H-function based image segmentation (Hsegment.m, [39]) and Sengupta et al’s. quantification of the number molecules in an RDF plot (RDFquant.m, [15]) have been included. All of these analyses can be performed using either 2D or 3D molecule position files. The use and dependencies of each function can be found in the header of each Matlab function. (ZIP) [file pcbi.1004634.s005.zip › help/MIiSR07.jpg]

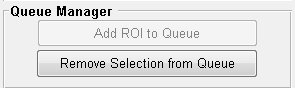

Supplement: S1 File — This zip-compressed archive contains the MIiSRconvert and MIiSR GUI’s, as well as stand-alone function for scripts for converting common super-resolution microscopy position files to Matlab-compatible matrices (fileConv.m), for cropping and filtering super-resolution microscopy position files (LoadCrop.m), and to perform the SAA (SAA2col.m, SAA3col.m), RDF and Ripley’s analysis (spatialStats.m), DBSCAN (DBSCAN.m), OPTICS (OPTICS.m) and hierarchal segmentation of the OPTICS plot (hierOPTICS.m) analyses described in this paper. In addition, stand-alone functions for Owen et al’s. Ripley’s H-function based image segmentation (Hsegment.m, [39]) and Sengupta et al’s. quantification of the number molecules in an RDF plot (RDFquant.m, [15]) have been included. All of these analyses can be performed using either 2D or 3D molecule position files. The use and dependencies of each function can be found in the header of each Matlab function. (ZIP) [file pcbi.1004634.s005.zip › help/MIiSR08.jpg]

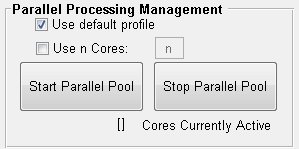

Supplement: S1 File — This zip-compressed archive contains the MIiSRconvert and MIiSR GUI’s, as well as stand-alone function for scripts for converting common super-resolution microscopy position files to Matlab-compatible matrices (fileConv.m), for cropping and filtering super-resolution microscopy position files (LoadCrop.m), and to perform the SAA (SAA2col.m, SAA3col.m), RDF and Ripley’s analysis (spatialStats.m), DBSCAN (DBSCAN.m), OPTICS (OPTICS.m) and hierarchal segmentation of the OPTICS plot (hierOPTICS.m) analyses described in this paper. In addition, stand-alone functions for Owen et al’s. Ripley’s H-function based image segmentation (Hsegment.m, [39]) and Sengupta et al’s. quantification of the number molecules in an RDF plot (RDFquant.m, [15]) have been included. All of these analyses can be performed using either 2D or 3D molecule position files. The use and dependencies of each function can be found in the header of each Matlab function. (ZIP) [file pcbi.1004634.s005.zip › help/MIiSR09.jpg]

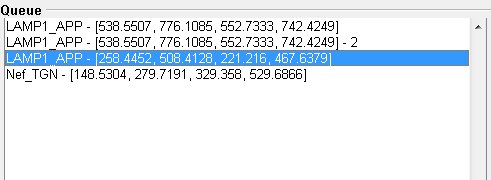

Supplement: S1 File — This zip-compressed archive contains the MIiSRconvert and MIiSR GUI’s, as well as stand-alone function for scripts for converting common super-resolution microscopy position files to Matlab-compatible matrices (fileConv.m), for cropping and filtering super-resolution microscopy position files (LoadCrop.m), and to perform the SAA (SAA2col.m, SAA3col.m), RDF and Ripley’s analysis (spatialStats.m), DBSCAN (DBSCAN.m), OPTICS (OPTICS.m) and hierarchal segmentation of the OPTICS plot (hierOPTICS.m) analyses described in this paper. In addition, stand-alone functions for Owen et al’s. Ripley’s H-function based image segmentation (Hsegment.m, [39]) and Sengupta et al’s. quantification of the number molecules in an RDF plot (RDFquant.m, [15]) have been included. All of these analyses can be performed using either 2D or 3D molecule position files. The use and dependencies of each function can be found in the header of each Matlab function. (ZIP) [file pcbi.1004634.s005.zip › help/MIiSR10.jpg]

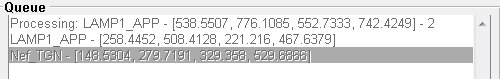

Supplement: S1 File — This zip-compressed archive contains the MIiSRconvert and MIiSR GUI’s, as well as stand-alone function for scripts for converting common super-resolution microscopy position files to Matlab-compatible matrices (fileConv.m), for cropping and filtering super-resolution microscopy position files (LoadCrop.m), and to perform the SAA (SAA2col.m, SAA3col.m), RDF and Ripley’s analysis (spatialStats.m), DBSCAN (DBSCAN.m), OPTICS (OPTICS.m) and hierarchal segmentation of the OPTICS plot (hierOPTICS.m) analyses described in this paper. In addition, stand-alone functions for Owen et al’s. Ripley’s H-function based image segmentation (Hsegment.m, [39]) and Sengupta et al’s. quantification of the number molecules in an RDF plot (RDFquant.m, [15]) have been included. All of these analyses can be performed using either 2D or 3D molecule position files. The use and dependencies of each function can be found in the header of each Matlab function. (ZIP) [file pcbi.1004634.s005.zip › help/MIiSR11.jpg]

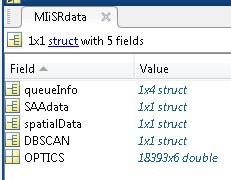

Supplement: S1 File — This zip-compressed archive contains the MIiSRconvert and MIiSR GUI’s, as well as stand-alone function for scripts for converting common super-resolution microscopy position files to Matlab-compatible matrices (fileConv.m), for cropping and filtering super-resolution microscopy position files (LoadCrop.m), and to perform the SAA (SAA2col.m, SAA3col.m), RDF and Ripley’s analysis (spatialStats.m), DBSCAN (DBSCAN.m), OPTICS (OPTICS.m) and hierarchal segmentation of the OPTICS plot (hierOPTICS.m) analyses described in this paper. In addition, stand-alone functions for Owen et al’s. Ripley’s H-function based image segmentation (Hsegment.m, [39]) and Sengupta et al’s. quantification of the number molecules in an RDF plot (RDFquant.m, [15]) have been included. All of these analyses can be performed using either 2D or 3D molecule position files. The use and dependencies of each function can be found in the header of each Matlab function. (ZIP) [file pcbi.1004634.s005.zip › help/MIiSRinfo.jpg]
