# Supplementary material for: MIiSR: Molecular Interactions in Super-Resolution Imaging Enables the Analysis of Protein Interactions, Dynamics and Formation of Multi-protein Structures
Source: PLoS Comput Biol. 2015 Dec 11;11(12):e1004634. doi: 10.1371/journal.pcbi.1004634 (PMC4676698; doi:10.1371/journal.pcbi.1004634)
Supplement: S1 File — This zip-compressed archive contains the MIiSRconvert and MIiSR GUI’s, as well as stand-alone function for scripts for converting common super-resolution microscopy position files to Matlab-compatible matrices (fileConv.m), for cropping and filtering super-resolution microscopy position files (LoadCrop.m), and to perform the SAA (SAA2col.m, SAA3col.m), RDF and Ripley’s analysis (spatialStats.m), DBSCAN (DBSCAN.m), OPTICS (OPTICS.m) and hierarchal segmentation of the OPTICS plot (hierOPTICS.m) analyses described in this paper. In addition, stand-alone functions for Owen et al’s. Ripley’s H-function based image segmentation (Hsegment.m, [39]) and Sengupta et al’s. quantification of the number molecules in an RDF plot (RDFquant.m, [15]) have been included. All of these analyses can be performed using either 2D or 3D molecule position files. The use and dependencies of each function can be found in the header of each Matlab function. (ZIP) [file pcbi.1004634.s005.zip › help/MIiSRconvertHelp.html]

Molecular Interactions in Super Resolution - Conversion Utility


# Molecular Interactions in Super Resolution (MIiSR) - Conversion Utility

---

### MIiSRconvert.m

This program is used to convert a variety of molecule position file formats from text-based files to files compatible with both Matlab and the MIiSR suite of analysis routines. The conversion utility can currently convert the following file formats:

- QuickPALM position files, saved in the default tab-delineated .xls file format
- Leica SR GSD position files, exported in the .ASCII format
- Tab-delineated text files formatted in one of four formats:
  - 2-column table, formatted as columns of X and Y positions (X/Y formatted)
  - 3-column table, formatted as above plus an intensity column (X/Y/I formatted)
  - 3-column tables, formatted as columns of X, Y and Z positions (X/Y/Z formatted)
  - 4-column table, formatted as above plus an intensity column (X/Y/Z/I formatted)

**Note:** Ideally, images should be acquired with the camera set on photon-counting mode, such that the 'intensity' reported in the source files indicates the number of detected photons rather than an arbitrary grey scale value.

**Note:** this program is provided as part of an academic publication. Please cite the following article if you use this software:

Fabiana A. Caetano, Brennan S. Dirk, Joshua H.K. Tam, P. Craig Cavanagh, Maria Goiko, Stephen S.G. Ferguson, Stephen H. Pasternak, Jimmy D. Dikeakos, John R. de Bruyn, Bryan Heit. MIiSR: Analysis of Molecular Interactions in Super-Resolution Imaging Enables the Study of Protein Interactions, Dynamics and Formation of Multi-protein Structures. 2015. *PLoS* *Computational Biology*.

---

### Starting and Configuring the Conversion Utility

To start the conversion utility, type "MIiSRconvert' into the Matlab command line, which will open the Conversion Utility GUI.

**Figure 1:** The MIiSr File Conversion Utility

The following variables must be set prior to adding files to the conversion queue:

**Bin Size:** This adjusts the size of the read/write blocks as files are processed. Larger Bin sizes typically result in faster file conversion, excessively large bin sizes can slow processing through consuming too much memory. Values of 8,000 - 12,000 typically provide the best performance, although smaller values (1,000 - 5,000) are better for computers with less memory, and larger values (15,000-20,000) are better for reading/writing data across a network. This is a global setting and cannot be altered for different files in the queue.

**Intensity Filter (Photons):** This setting removes molecules from the dataset which were detected with fewer than the entered number of photons. Low photon numbers degrade the precision of the resulting image. The estimated precision of the photon cut-off is automatically propagated to the 'Precision' intensity filter. To prevent filtering set this value to zero.

**Intensity Filter (Precision):** This setting removes molecules from the dataset which were detected with a precision greater than the entered precision. The precision cut-off is automatically converted to the number of photons required for that degree of precision and propagated to the 'Photons' intensity filter. To prevent filtering set this value to zero.

**Scale (nm/pixel):** This setting will convert position files scaled in units of pixels to files scaled in nanometres. If the position files are pre-scaled to nanometres, set this value to 1.

**Intensity to Photon Scaling:** All intensity filtering is performed on the number of detected photons. If the image series was not captured using the camera set in photon-counting mode, a scaling value can be entered into this window to convert grey scale intensity into photons. Set this value to 1 to prevent intensity scaling.

---

### Adding Files to the Queue

Files of different formats can be added to the queue, and different intensity filtering and scaling values can be applied to each file.

**To add a single file:** First, make sure the correct filtering and scaling values are entered in the "Conversion Settings" panel, then select the desired file type from the *Input File Format* drop-down menu. Next, click the "Add Single File" button. Browse to the location of the file you wish to add, and select the file to be converted, and click 'Open'. The file will then be added to the queue, with the filtering and scaling options indicated in the "Conversion Setting" panel.

**Figure 2:** Input File Format drop-down menu.

**Figure 3:** Adding a single QuickPALM file to the queue.

**To add all files in a single folder:** First, make sure the correct filtering and scaling values are entered in the "Conversion Settings" panel, then select the desired file type from the *Input File Format* drop-down menu. Next, click the "Add Single Folder" button. Browse to the folder you wish to add, and click the "Select Folder" button.. All files with the appropriate file extension in the folder will then be added to the queue. The same filtering and scaling options (as indicated in the "Conversion Setting" panel) will be applied to all files.

**Figure 4:** Adding a single folder containing X/Y formatted tab-delineated text files to the queue.

**To add all files in a folder and all sub-folders:** First, make sure the correct filtering and scaling values are entered in the "Conversion Settings" panel, then select the desired file type from the *Input File Format* drop-down menu. Next, click the "Add Folder & Subfolder" button. Browse to the root folder you wish to add, and click the "Select Folder" button. The software will then search the folder and all subfolders for files with the appropriate file extension and add them to the queue. The same filtering and scaling options (as indicated in the "Conversion Setting" panel) will be applied to all files.

---

### Managing the Queue

**Figure 5:** File queue

Files in the queue can be deleted using the 'Delete Entry" button at the bottom of the queue. If a file was added to the queue with the wrong filtering or scaling information it must be deleted and re-added to the queue. Clicking the 'Start' button in the main window will begin the processing of the files in the queue; the conversion utility interface will become inactive at this time, and will close automatically when processing is complete. As files are converted they will be removed from the queue. Do not close the conversion utility window during processing as this will interrupt the conversion process.
