# Supplementary material for: MIiSR: Molecular Interactions in Super-Resolution Imaging Enables the Analysis of Protein Interactions, Dynamics and Formation of Multi-protein Structures
Source: PLoS Comput Biol. 2015 Dec 11;11(12):e1004634. doi: 10.1371/journal.pcbi.1004634 (PMC4676698; doi:10.1371/journal.pcbi.1004634)
Supplement: S1 File — This zip-compressed archive contains the MIiSRconvert and MIiSR GUI’s, as well as stand-alone function for scripts for converting common super-resolution microscopy position files to Matlab-compatible matrices (fileConv.m), for cropping and filtering super-resolution microscopy position files (LoadCrop.m), and to perform the SAA (SAA2col.m, SAA3col.m), RDF and Ripley’s analysis (spatialStats.m), DBSCAN (DBSCAN.m), OPTICS (OPTICS.m) and hierarchal segmentation of the OPTICS plot (hierOPTICS.m) analyses described in this paper. In addition, stand-alone functions for Owen et al’s. Ripley’s H-function based image segmentation (Hsegment.m, [39]) and Sengupta et al’s. quantification of the number molecules in an RDF plot (RDFquant.m, [15]) have been included. All of these analyses can be performed using either 2D or 3D molecule position files. The use and dependencies of each function can be found in the header of each Matlab function. (ZIP) [file pcbi.1004634.s005.zip › help/MIiSRHelp.html]

Molecular Interactions in Super Resolution


# Molecular Interactions in Super Resolution (MIiSR)

---

### MIiSR.m

This program is used to manage the analysis of a series of molecular interaction and image segmentation algorithms on a super-resolution image set. These analyses and procedures include:

1. Generation of cropped single-colour and RGB images from single-colour position files.
2. Density filtering of the image
3. Spatial Association Analysis of molecular interactions between up to 3 different molecular species
4. Spatial Statistical Analyses of co-clustering
5. Cluster identification by DBSCAN or OPTICS image segmentation

Multiple regions of interest (ROI's) can be assigned to a single image, and multiple images can be run in a single queue. Note that the super-resolution position files analysed by this program must first be converted to a Matlab-compatible matrices using the included MIiSRconvert utility, which can be started by typing 'MIiSRconvert' into the Matlab command line. Instructions on the use of this utility can be found in the conversion utility's help file.

**Note:** this program is provided as part of an academic publication. Please cite the following article if you use this software:

Fabiana A. Caetano, Brennan S. Dirk, Joshua H.K. Tam, P. Craig Cavanagh, Maria Goiko, Stephen S.G. Ferguson, Stephen H. Pasternak, Jimmy D. Dikeakos, John R. de Bruyn, Bryan Heit. MIiSR: Analysis of Molecular Interactions in Super-Resolution Imaging Enables the Study of Protein Interactions, Dynamics and Formation of Multi-protein Structures. 2015 *PLoS* *Computational Biology*.

---

### Starting MIiSR & GUI Overview

To start the MIiSR utility it must be installed in a path accessible by Matlab. Once installed, type "MIiSR' into the Matlab command line, which will open the Conversion Utility GUI. This will open the MIiSR utility:

**Figure 1:** MIiSR Utility, immediately after start-up

The utility is divided into three columns, each controlling a different aspect of the analysis process. The left column allows the user to load images and define ROI's. The middle column allows the user to control the analyses performed and to manage parallel processing. The right column controls displays the queue of analysis jobs to be performed, and once analysis is started, indicates progress.

The workflow for the utility is to use the left column to load an image for analysis and select a specific ROI upon which analysis will be conducted. Once an ROI is selected, the middle column is used to determine which analyses are to be performed on the ROI, configure each analysis procedure, and then add the ROI & its analyses to a processing queue. This process can be repeated for multiple ROI's from a single image, and for multiple images. Different analytical options can be selected for each ROI/image. Once all images/ROI's are loaded onto the queue (right panel), processing can be started with the 'Start Queue' button; progress of the analysis is indicated in the queue.

---

### Channel Panel

The *Channel* panel allows the user to select the image files for each colour channel in the image, and to assign a name to each channel. The name assigned to each panel will be used in subsequent file and folder names, as well as to label axes in graphs produced by the various analysis routines.

**Figure 2:** Channel panel

To load the first channel, click the 'Add Channel 1' button, and use the file selection window to browse to the folder containing the converted (using MIiSRconvert) super-resolution position file, and select the desired file. Channel is automatically assigned a red colour in the "Select ROI" window. At this time a descriptive name should be entered into the "Name for Channel 1" text box.

Once the first channel is loaded the 'Add Channel 2' button will become active. This channel is added in the same fashion as channel 1, and will appear green in the "Select ROI" widow. After adding the second channel, the 'Add Channel 3' button will become active, with the channel displayed in blue in the 'Select ROI' window. The addition of the second and third panel are not required, although not all analyses are possible with single-colour images.

---

### Select ROI Panel

The *Select ROI* panel allows the user to load the positions files selected in the "Channel Panel" into a preview image, and then to select ROIs for further analysis on this image.

One the files for each colour channel is selected, and a name for each channel entered, the image preview can be loaded by clicking the 'Load Image' button located at the bottom of the Select ROI panel.

After loading the image an ROI can be selected by clicking the 'New ROI' button. Left-click on the image at one corner of the desired ROI, then drag the mouse to expand the ROI. Upon releasing the left mouse button, the ROI's edges can be fine-tuned by dragging them with the mouse, and the ROI moved on the image by dragging the centre of the ROI. Once the desired ROI size and position is complete, double-click in the centre of the ROI to activate it.

**Important Note:** The SAA, Ripley's and RDF analysis will not produce accurate results if the ROI extends beyond the edge of the cell. As such it is critical that all ROI's used for these analyses are located entirely within the body of the imaged cell/object.

The current ROI can be cleared using the "delROI' button. To clear the ROI and image, click on the Clear Image button. In both of these cases, any analyses added to the queue will not be removed, allowing these buttons to be used to add multiple ROI's to the analysis queue from a single image, and to add ROI's from multiple images, to the analysis queue.

**Figure 3:** Select ROI panel, displaying a 2-channel image and an active ROI.

---

### Density Filter Panel

The density filter panel allows for filtering of the image, where in molecules located in areas below a set density level are removed. This filter functions by determining the regional density in an area of radius determined by the 'Filter Density' textbox. Molecules in each channel are removed if they are in a local region of density equal to the median density of the image plus X standard deviations, where X is the value entered into the *Ch1/2/3* textboxes. For example:

- Ch1 = 0, Filter Distance = 100: Any molecules in channel 1 found in a 100 nm radius local region with less than the median density of channel 1 will be removed.
- Ch1 = 1, Filter Distance = 50: Any molecules in channel 1 found in a 50 nm radius local region with less than (median + 1 SD) the median density of channel 1.
- Ch1 = -0.5, Filter Distance = 200: Any molecules in channel 1 found in a 200 nm radius local region with less than (median - 0.5 SD) the median density of channel 1.

Leaving any of the Ch1/2/3 textboxes empty, or with '[]' entered, will prevent filtering of that channel.

**Important Note:** This filtering is non-linear and preferentially excludes non-clustered molecules from the data set. As such it should not be used for SAA or Spatial Statistics under most circumstances. This filtering method can greatly improve DBSCAN and OPTICS segmentation.

**Figure 4:** Density Filter panel.

---

### Spatial Association Analysis Panel

The Spatial Association Analysis panel controls SAA analysis of the image. This analysis quantifies intermolecular interactions between two or three image channels. The 'Perform SAA' checkbox will determine if SAA analysis is performed on the current ROI. If selected the user must define the following analysis options:

- Maximum analysis distance (nm): Maximum intermolecular separation distance to be considered for SAA analysis. This value should be set smaller for higher molecular density images, and larger for lower molecular density images.
- Randomization cycles: Number of random images generated, used to determine if the degree of observed intermolecular interactions are statistically significant.
- Analyzed fraction: Portion of molecules to be analysed. Setting this below 1 will analyze a randomly selected subset of molecules in the image. Performing analysis on a subset of molecules is beneficial in images containing high levels of over-sampling.
- Estimate Randomization Area: If checked, MIiSR will attempt to refine the randomization area by finding a polygon which encloses all points while minimizing molecule-free area(s) included in the ROI. This feature acts to find putative "invisible" (unlabeled) structures within the user-selected ROI.
- Colocalization Criterion Cutoff (CDC) calculations - these can be calculated automatically, or be user-set

- Calculate CDC checkbox - automatically calculate CDC

- Standard Deviations: Standard deviations of the microscopes precision to be considered when calculating the CDC; smaller values set a more stringent CDC but risks excluding *bona fide* molecular interactions. Values of 1.65-2.0 are typical.
- Registration Error: Value added to the calculated CDC, used to account for measured chromatic aberration in the microscope system.

- User-Defined CDC: this option must be used for microscope systems which do not provide data on the precision of fluorophore detection. Appropriate values are dependent on the microscope system and fluorophore brightness, but values of 25-30 and 28-36 are typical for 2- and 3-colour images respectively.

**Figure 5:** Spatial Association Analysis panel.

---

### Spatial Statistics Panel

The Spatial Statistics panel performs co-clustering analysis on one or two colour channels. The user selects whether Ripley's and/or Radial Distribution Analysis (RDF) should be performed, sets a maximum intermolecular distance to be considered in the analysis, and defines which channels are to be compared\*.

\*For single-channel analyses, 'Channel 1' should be selected as both the *Primary* and *Secondary* channel.

**Figure 6:** Spatial Statistics panel.

---

### Clustering Analysis Panel

The clustering analysis panel allows for segmentation of clusters in a single image channel - i.e. individual clusters of molecules, as well as the edges of each cluster, will be computationally identified. The resulting clusters can then be analysed further, with user-prepared scripts, for features such as cluster area, density, etc.

***DBSCAN******:*** If selected, the user must select a minimum cluster size (in number of molecules) and channel to process. Optionally, a neighbourhood radius for calculations of local density can also be defined. If the latter is left empty, the software will attempt to predict this value based on the median density of the image.

***OPTICS:*** If selected, the user must select a minimum cluster size (in number of molecules) and channel to process. Unlike DBSCAN, OPTICS does not provide a direct output of clusters, and instead produces a reachability distance (RD) plot from which clusters can be segmented through a variety of approaches. One form of automated segmentation is included, and can be run by selecting the 'Hierarchical Segmentation' option. If selected, a value must be set for the Peak Ratio; the minimum ratio between the reachability value of a split point in the RD plot and the mean value of each resulting cluster to be considered a statistically significant split. Values of 0.5 to 0.8 are typical. A detailed explanation of this value can be found in the supplemental materials section of our paper.

**Figure 7:** Clustering Analysis panel.

---

### Queue Manager

Once an ROI has been selected and the analyses for that ROI configured, the ROI and its attached analyses need to be added to the analysis queue. This is done by selecting the 'Add ROI to Queue' button in the Queue Manager panel.

Once this is completed the analysis setting can be changed for the existing ROI and the ROI + new analysis settings added to the queue with the 'Add ROI to Queue' button. Likewise, a new ROI can be added by deleting the old ROI and establishing a New ROI using the respective buttons in the 'Select ROI' panel. Once the analysis settings are configured appropriately, the new ROI can be added to the queue with the 'Add ROI to Queue' button. Finally, the existing image and ROI can be cleared with the 'Clear Image' button in the Select ROI panel, a new image loaded, and ROI's from the new image added to the queue with the 'Add ROI to Queue' button.

Items on the queue can be removed by selecting the item to be deleted in the queue, and then clicking the 'Remove Selection from Queue' button in the 'Queue Manager' panel.

**Figure 8:** Queue Manager Panel

---

### Parallel Processing Management Panel

To maximize processing speed, many of the included analyses utilize parallel processing. This is managed using the Parallel Processing Management panel. Generally, the default profile auto-configured when Matlab is installed should be used, but under some circumstances users may wish to manually configure parallel processing - i.e. for very large images, using a smaller number of cores may speed processing by providing a larger amount of memory per core. To prevent parallel processing, a manual value of 1 must be entered.

- To use the default profile, select the 'Use default profile' checkbox.
- To use a defined number of cores, select the 'Use n Cores' checkbox and enter the desired number of cores in the 'n' textbox:

- n = 0: Default profile will be used
- n = 1: No parallel processing
- n > 1: Use indicated number of cores (decimal values will be rounded up to the nearest integer)

**Note:** Although any value can be used for the number of cores, using more than the number of physical cores available to the computer should be avoided as this will greatly decrease computational performance.

**Note:** The user interface will become inaccessible while starting or stopping parallel processing.

**Figure 9:** Parallel Processing Manager

---

### Queue

The queue displays the ROI's added to the queue for processing, and during processing, indicates progress. Items in the queue are listed by channel names, followed by the ROI coordinates in square brackets. If the same ROI is being analysed with different analysis settings, a number will be appended to the name in the ROI (e.g. '- 01', '- 02', etc). Highlighted items will be removed from the queue if the "Remove Selection from Queue' button in the Queue Manager panel is clicked.

Clicking the 'Start Queue' button at the bottom of the queue will begin analysis of all items in the queue. Additional items cannot be added to the queue once processing has begun, so this should not be done until all items are added to the queue.

**Note:** If parallel processing is not started prior to clicking the 'Start Queue' button, parallel processing will automatically be started using the settings defined in the Parallel Processing Manager.

**Figure 10:** The queue. In this example the first two entries are the same ROI analysed with different analysis settings, and the third item is a different ROI in the same image as the first the first two items. The third entry is an ROI from a new image.

---

### Data

Once the 'Start Queue' button is clicked analysis will begin. During this time the computer should not be used for other purposes as this can interfere with graphing. For each item on the queue a folder will be created with the same name as the queue entry; this folder will be located in the same folder as the position file for channel 1, and all graphs and raw data for the ROI will be saved in that folder.

**Figure 11:** The queue during processing. The first item has completed processing and has been removed from the queue. The second item is being processed, as indicated in the queue.

For each ROI the following will be saved regardless of analysis options selected. 'Filename' indicates the name of the analysis, as indicated in the queue:

- TIFF images of the original image and ROI, files are named 'Filename.tif' and 'Filename - Cropped.tif' respectively.
- MIiSRdata - Filename.mat: File containing all raw data from the selected analyses; the exact contents of this are described in detail, below
- Graphs: Graphs of data are saved in matlab .fig files, and as PDF's in select cases. These files are named as <analysis type> - <Filename> - <Details>.fig or .pdf. For example, fold-enrichment graphs from SAA analysis would be saved as 'SAA - Filename - Fold Enrichment.fig'

All of the data contained in these graphs, as well as other data potentially of use, is saved in the MIiSRdata file. When loaded this file contains two variables:

- Conditions: this structure contains all information used by the sub-scripts for analysis of the data. This variable can be passed to these functions to manually re-process the data using the same settings
- MIiSRdata: a structure containing the results of all analyses; the exact contents of which will be determined by the analysis options selected for that ROI

**Figure 12:** The MIiSRdata structure, displaying all possible entries. Each entry is described below.

The following data can be found in each of the MIiSRdata variables:

- queueInfo: Settings used for all analyses of the current ROI
- SAAdata: data from the SAA analysis. A full description of all outputs can be found in the header of SAA2col.m and SAA3col.m, but key values are:

- SAAcutoff: the CDC used for analysis
- distTableIm1/2/3: Distance to nearest neighbour tables for channels 1, 2 and 3
- BarData/eBarData/fBarData: Bar graph values in absolute numbers of interacting molecules, enrichment compared to random, and fold-random

- Spatialdata: Data from spatial statistics:

- Kr: Ripley's K(r) values (column 1) and derivative of K(r) (column 2)
- Lr: Ripley's L(r) values (column 1) and derivative of L(r) (column 2)
- Hr: Ripley's H(r) values (column 1) and derivative of H(r) (column 2)
- Gr: RDF values
- Xr: Radius values (in nm), for each row of the Ripleys and RDF tables

- DBSCAN: DBSCAN segmentation data

- k: Minimum cluster size used by DBSCAN algorithm
- Eps: Neighbourhood radius used by DBSCAN algorithm
- Noise: table of X/Y/Z positions of all molecules not assigned to a cluster
- Cluster: structure containing X/Y/Z coordinates and edge points for each identified cluster

- OPTICS: OPTICS segmentation data

- 6 x *n* table, each row corresponding to a single molecule, each column corresponding to:

- Column 1: Order of molecule *n* (position of molecule *n* in pre-segmentation molecule position file)
- Column 2: Reachability Distance of molecule *n*
- Column 3: Core Distance of molecule *n*
- Columns 4/5/6: X/Y/Z coordinates of molecule *n*

- OPTICSsegment: OPTICS segmentation structure. A full description can be found in the header of the hierOPTICVS.m file. Key features include

- Parent: cluster entry which is the parent to the current cluster
- l/rChild: cluster entries corresponding to the left and right children of the current cluster. Entries will be empty if current cluster is a leaf (terminal cluster)
