# Supplementary material for: MIiSR: Molecular Interactions in Super-Resolution Imaging Enables the Analysis of Protein Interactions, Dynamics and Formation of Multi-protein Structures
Source: PLoS Comput Biol. 2015 Dec 11;11(12):e1004634. doi: 10.1371/journal.pcbi.1004634 (PMC4676698; doi:10.1371/journal.pcbi.1004634)
Supplement: S1 Text — Complete derivation of all algorithms used in the MIiSR toolbox. (DOCX) [file pcbi.1004634.s006.docx]

# S1 - Supplemental Text

## The Spatial Association Analysis (SAA) Algorithm

The SAA algorithm functions by first measuring the Euclidian distance from each molecule in the first color channel to the nearest neighboring molecule(s) in the other color channel(s). So for a molecule in the first color channel (*p*), the distance to the nearest neighbor molecule in the second color channel (*q*) would be:

$dist\left( p,q \right)= \left( \Delta x^{2}+ \Delta y^{2}+\Delta z^{2} \right)^{-½}$ (1)

Where Δx, Δy and Δz indicate the separation of the molecules along the x, y and z axis respectively.

The resulting table of inter-molecular distances is then binned into a histogram; the bin-size is user defined, but bins of approximately ½ the lateral precision of the super resolution microscope (typically 10-20 nm for GSDM and PALM microscopes) work well. Next, the fraction of potentially interacting molecules is determined by determining the fraction of molecules in color channel 1 with nearest neighbors in the other color channel(s) separated by a distance less than the CDC (see next section, equation 4). It is important to note that this interaction is non-symmetrical, and thus the observed fraction of interacting molecules comparing channel 1 to channel 2 will be different than if channel 2 is compared to channel 1.

The above calculation identifies the number of potentially interacting molecules in the image, but does not indicate whether the observed number of interactions is greater than that expected due to chance colocalization between non-interacting molecules. Thus, it is necessary to take a Monte Carlo approach [1] to model non-interacting molecules in order to determine whether the SAA-detected interactions represent true intermolecular interactions, or chance interactions of non-associated molecules. In this approach the limits of the area (for 2D images) or volume (for 3D images) is determined by identifying the bounding box of the image, defined by the minimum and maximum *x*, *y* and *z* coordinates of molecules in the image. Next, the positions of the molecules in color channel 1 are randomized within this area/volume, producing a model of non-interacting (i.e. randomly distributed) molecules, and the SAA measure repeated utilizing the randomized channel. It is not necessary to randomize all color channels, as randomization of the primary channel alone provides the same SAA result as randomization of all color channels (data not shown). Generally, this randomization process is repeated 10 to 100 times, and the results averaged, in order to ensure that the Monte Carlo simulations produce a statistically representative sample of non-interacting molecules.

In SAA analysis, molecules undergoing *bona fide* interactions will show a statistically significant increase in the fraction of molecules separated by less than the CDC in the real images compared to the Monte Carlo simulations, while molecules undergoing exclusion will have a statistically significant decrease in the fraction of molecules separated by less than the CDC in the real images compared to the Monte Carlo simulations.

## Derivation of the Colocalization Distance Criterion

The cutoff used for SAA analysis, termed the Colocalization Distance Criterion (CDC), must take into account the precision of fluorophore detection, the desired level of statistical certainty of detecting interactions, and any image registration defects of the microscopy system used for imaging. The error induced by the limited precision of super-resolution microscopy systems is determined by the root-mean-squared error of the precision of the microscope, which for a given channel (σ_c_) is determined by the number of photons collected. For an image containing *m* particles, this can be approximated from the width of the point-spread function *ω* (typically 200-250 nm for a high numerical aperture lens) and the number of collected photons for each particle *N_i_*:

$\sigma_{c}=\frac{\sum_{i=1}^{m} \frac{\omega}{\surd N_{i}}}{m}$ (2)

Because we are assessing multiple fluorophore channels, the cross-channel error for an image containing *n* channels is determined by calculating the root mean square error (σ_RMS_) of the positional precision of all channels being assessed:

$\sigma_{RMS}=\left( \sum_{i=1}^{n} \sigma_{ci} \right)^{\frac{1}{2}}$ (3)

The result of equation 3 is then multiplied by a 90% or 95% probability cut-off (1.65 or 2.0 standard deviations respectively). To this is added the sum of any chromatic aberration or cross-channel image registration inaccuracy (I_reg_), determined experimentally [2] to produce the Colocalization Distance Criterion (CDC), the distance below which molecules are considered to be potentially interacting:

$CDC=1.65\sigma_{RMS}+I_{reg}$ (4)

It is important to note that optical aberrations or defects in image registration can lead to excessively large values of the CDC. One advantage of the statistical approach employed in SAA analysis is that this aberration/registration defect-created increase in the CDC does not measurably increase the rates of false positives or false negatives in SAA analysis (data not shown). However, not accounting for these defects when calculating the CDC can increase the rate of false negatives, through excluding interacting molecules artificially separated by these defects (data not shown). As such, it is important to accurately quantify image registration and optical aberration defects prior to engaging in SAA analysis, and many robust methods have been published for assessing these defects (e.g. [2]). Lastly, the accuracy to which intermolecular distances can be measured is limited to ~⅓^rd^ the CDC (Fig. 1B), but the accuracy of these measurements will decrease dramatically in the presence of aberration or image registration defects.

## Ripley’s K & H Function

Ripley K function represents an easier-to-interpret measure of clustering than RDF analysis, but requires the use of molecule position files in place of images and thus is computationally intense compared to RDF analysis (Fig. S3, [7]). The K-function quantifies the average number of molecules within a radius *r* of a given particle, calculated by summing the number of particles with a Euclidian separation *d_ij_* less than *r*, normalized to the average particle density in the image ρ and the total number of particles in the image *n*.

$K\left( r \right)=\rho^{-1}\cdot\sum_{i\neq j} \left( d_{ij}<r \right)/n$ (6)

In MIiSR, a table of distances from each molecule to all other molecules in the data set is generated, which is then binned into a histogram of molecule numbers at increasing distance. The K function (Kr) is calculated iteratively from this histogram (tMat), along with a count of the total number of molecules in the image (N) and the average molecular density (lambda, in units of molecules/nm^2^):

| %K Calculation  for kk=1:length(tMat)  Kr(kk,1) = sum(tMat(1:kk))/N;  end  Kr = Kr./lambda; |
| --- |

For unclustered data, the K function returns the area of a circle of equivalent radius, thereby producing a curve proportional to πr^2^. In contrast, clustered data exhibits higher K values at small radii due to above-average molecular density at short distances, and lower K values at large radii due to below-average particle densities between clusters (Fig. S3A). These plots are hard to interpret, and as such the K function is usually normalized to area (L function) or area and radius (H function):

$L\left( r \right)=\left( {K\left( r \right)}/\pi\right)^{-½}$ (7)

$H\left( r \right)=L(r)-r$ (8)

In MIiSR, these values are calculated directly from the K function and the radius (Xr):

| % L and H Calculation  Lr = sqrt(Kr./pi());  for ii=1:length(Xr)-1  Hr(ii,1)=Lr(ii)-(Xr(ii));  end |
| --- |

For unclustered data the H(r) plot will have a value of 0 at all radii, while clustered data produces a peaked curve. The average cluster radius is equal to the value of the radius at the maximum value of H(r), while increasing peak height indicates a larger portion of molecules undergoing clustering (Fig. S3A). Mean inter-cluster separation is equal to two times the radius where H(r) re-crosses the zero line. Non-uniform data that otherwise lacks significant co-clustering will display above-zero values of H(r), but will not drop below H(r) = 0 at larger radii [7].

## The Radial Distribution Function (RDF)

The RDF, also known as the pair-correlation and G functions, describes how density varies as a function of distance from a reference molecule [4,5]. Specifically, the RDF quantifies the probability of finding particles within a torus of radius *r* and thickness *d_r_* of another particle in a field of given density ρ:

$G\left( r \right)=2\pi\rho rd_{r}$ (5)

The average cluster radius and separation distance for regularly sized and spaced clusters can be determined from the width of the first peak of G(r) and the spacing between subsequent peaks, respectively (Fig. S2A, [6]). However, the more heterogeneous nature of biological samples often produces RDF curves without secondary peaks, thus limiting analysis to average cluster radius (determined by the width of the first peak, Fig. S2A) and relative measures of the total fraction of clustered molecules, which is indicated by a downward shift of the G(r) plot [6]. Unclustered samples will result in G(r) ≈ 1 at all values of *r* (Fig. S2A, B, [6]). In MIiSR the RDF is calculated using the average molecular density (lambda), radius (Xr), the histogram of molecular distances (tMat), and the width of the bins in the histogram (BinW), all determined previously for calculation of the K function:

| %G(r) (RDF) Calculation  for ii=1:length(tMat)  Gr(ii) = tMat(ii)/(2*pi()*lambda*BinW*(Xr(ii+1)));  end |
| --- |

## Density-Based Spatial Clustering of Applications with Noise (DBSCAN) Algorithm

DBSCAN is a segmentation algorithm which identifies clusters in an image based on the principal of density reachability [8,9]. The particles *p* and *q* are considered density reachable if they meet one of two criteria: 1) *p* and *q* are considered to be *directly density-reachable* if *q* is within a distance ≤ ε of *p*, and *p* has at least *k* neighboring molecules within a distance of ε, or 2) *p* and *q* are considered to be *density-reachable* if they are separated by a distance greater than ε but are connected by a series of molecules which form of chain of directly density-reachable molecules (Fig. S4A). Specifically, in an image containing the set of *D* molecules, the ε-neighborhood of molecule *p* contains the set of molecules:

$N_{\varepsilon}(p)=\left\{ q\in D | dist(p,q)\leq\varepsilon\right\}$ (8)

Molecule *q* is *directly density-reachable* from molecule p if the following criteria are met:

$q \in N_{\varepsilon}\left( p \right)$ AND $\left| N_{\varepsilon}\left( p \right) \right|\geq k$ (9)

Alternatively, molecules *p* and *q* are *density-reachable* if connected by a chain of molecules where (P_1_, … , P_n_), where P_1_ = *p*, P_n_ = *q* and such that P_i+1_ is directly density-reachable from P_i_. Any point which is neither directly density reachable nor density reachable is considered to be unclustered (i.e. noise).

It is important to note that density reachability is non-symmetrical, meaning that point *q* can be directly density-reachable from point *p*, but point *p* may not be directly density reachable from point *q*. This occurs when point *p* is at the edge of a cluster, and thus will not satisfy Nε(*p*) ≥ *k*, while point *q* would be in the core of the cluster and thus satisfy Nε(*q*) ≥ *k*. This asymmetry enables DBSCAN to not only identify clusters in images, but also imparts the unique ability to identify edge versus core points within the clusters (Fig. S4A, B):

- The molecule *p* is considered a *core point* if Nε(*p*) ≥ *k*
- The molecule *p* is considered an *edge point* if Nε(*p*) < *k*, but molecule *p* is directly density-reachable from another point.

Because both the neighborhood size (ε) and minimum cluster size (*k*) are static values, successful use of DBSCAN is limited to images where clusters have similar molecular density (Fig. 3B, Fig. S4B). Moreover, DBSCAN requires that the user provide reasonable values for both *k* and ε – parameters which are not always known *a priori*.

## Ordering Points To Identify the Clustering Structure (OPTICS) Algorithm

OPTICS was designed to overcome the limitation imparted on DBSCAN by the use of a static neighborhood size (ε). OPTICS requires the user only define the minimum number of particles to be considered a cluster (*k*), and then defines the size of the each molecules neighborhood based on measuring the *Reachability Distance* (RD) for each molecule in the image [10–12]. The OPTICS algorithm begins by creating a list of the positions of all molecules in the image, ordered such that neighboring entries in the list represent molecules which are nearest neighbors in the image. This list is then processed sequentially, starting with the first molecule in the list (*p_i_*, where i=1). To calculate RD two measures must be made – the first is the *core distance* (CD), defined as the Euclidian distance from *p_i_* to the *k*^th^ closest molecule. Secondly, the Euclidian distance from *p_i_* to *p_i+1_* is measured. RD is defined as the larger of these two values:

$CD\left( p_{i} \right)=k^{\mathrm{th}} smallest distance to N\varepsilon\left( p_{i} \right)$ (10)

$RD\left( p_{i} \right)= \max\left\{ \mathrm{CD}\left( p_{i} \right), \mathrm{dist}(p_{i}, p_{i+1}) \right\}$ (11)

The first molecule is then marked as processed, and molecules *p_i+1_*…*p_i=n_* processed sequentially. The final output of the OPTICS algorithm is thus an ordered list of molecules annotated by their reachability distance. This data is then plotted as a reachability plot, wherein the points are ordered along the *x*-axis and the reachability distance plotted on the *y*-axis (Fig. S4C-E). Clusters appear as “valleys” in the reachability plot; the “deeper” the valley the more dense the cluster. Unlike DBSCAN, OPTICS can also find clusters within clusters, as these “sub-clusters” will appear as smaller/deeper valleys inside of the larger/shallower valley of the super-cluster (Fig. S4D, [10]).

In MIiSR, CD is calculated iteratively for each molecule in the data set (pXY, where pXY is a m x 3 matrix of X/Y/Z coordinates) by calculating the distance from the current point to all subsequent points, ordering the resultant distance table by distance, and defining the CD as the k^th^ closest point (where k is the user-set minimum cluster size):

| % Generate CD, RD & order tables  RD = zeros(m,1);  CD = RD;  parfor ii=1:m  tPos = pXY(:,1:3); %extract x/y/z coordinates  tPos(ii,:) = []; %eliminate self-position  D = sort(sqrt((tPos(:,1)-pXY(ii,1)).^2 + (tPos(:,2)-pXY(ii,2)).^2 + (tPos(:,3)- . . .  . . . pXY(ii,3)).^2));  CD(ii)=D(k-1); %CD as most distant particle in local cluster of size k  end |
| --- |

In MIiSR, RD is calculated sequentially for each molecule in the data set (pXY) by calculating the distance from the currently processed molecule to its nearest unprocessed neighbor, and recording the larger of either this value, or CD, as the RD:

| % Calculate RD  for ii=1:(m-1)  [RD(ii+1), nnD] = min(sqrt((pXY(ii+1:end,1)-pXY(ii,1)).^2 + (pXY(ii+1:end,2)- . . .  . . . pXY(ii,2)).^2 + (pXY(ii+1:end,3)-pXY(ii,3)).^2)); %distance to remaining points  tPos = pXY(ii+1,:); %prepare to rearrange dataset  pXY(ii+1,:) = pXY(ii+nnD,:); %move closest particle to below current particle  pXY(ii+nnD,:) = tPos;  RD(ii+1) = max([RD(ii+1), pXY(ii+1,5)]); %set RD  end |
| --- |

Extracting clusters from the OPTICS RD plot is more difficult than with DBSCAN. The simplest method of cluster extraction is to pick a set RD threshold, and to then define clusters as valleys in the RD plot divided by peaks which exceed the threshold. Although simple to implement, this method imparts a static neighborhood size on cluster detection, thus providing an output little different than that of DBSCAN. Several other methods have been proposed to separate clusters based on variable RD values. The original implementation of the OPTICS algorithm proposed two methods – manual (visual) identification of valleys, and a less subjective method based on identifying the edges of clusters as “steep drops” in the reachability plot [10]. Although the steepness measures provides an automated method for extracting clusters, it still requires the selection of largely arbitrary “steepness” values and is highly subjective to both noise and the value of *k* selected for the initial OPTICS analysis [11,12].

Daszykowski *et al.* [11] proposed a method which, when applied to microscopic images, would identify clusters based on correlating RD’s with the intensity of each detected fluorophore. Clusters would be identified as low-points in the reachability plot that have a high intensity value. While this method may work for super-resolution methods that rely on structured illumination (e.g. STED and SSIM microscopy [13,14]), we found that there was no correlation between the RD and the intensity of individually detected fluorophores in GSDM imaging, likely due to the stochastic nature of the imaging process (data not shown).

In this toolbox we have utilized a variation of the method proposed by Sander *et al.* [12]. This method hierarchally breaks down the RD plot, producing a nested dendogram of identified clusters within the dataset (Fig. S4C-D). This hierarchal breakdown not only allows for detection of individual clusters independent of their RD values (i.e. independent of cluster density), but also readily identifies sub-clusters within larger clusters. This algorithm begins by finding the highest RD value in the RD plot, and then divides the RD plot into “left” and “right” segments centered on this peak. The average RD for the left and right segments is calculated, and if found to be sufficiently less than the RD value of the dividing peak (this value is user-defined, but 75% is typical), the division is considered significant. The left and right segments are then split into two segments, with each split centered on the highest RD value found within the respective segment. This hierarchal breakdown of the image is continued until segments are reached that are either smaller than a user-defined minimum cluster size, or where a split does not create segments with average RD’s sufficiently smaller than the dividing peak to be considered a significant division.

The one limitation of OPTICS compared to DBSCAN is the lack of defining edge versus core molecules within clusters. A number of approaches can be taken to identify the bounding area of a cluster. This can be as simple as defining the bounding box of the cluster based on the minimum and maximum *x*, *y* and *z* coordinates of molecules within the cluster, or more sophisticated edge-tracing algorithms can be applied. We have employed convex hull analysis to define the bounding edge of clusters in our hierarchal analysis tool hierOPTICS.m, which provides a much tighter boundary to the cluster than defining a bounding box. However, convex hull calculations tend to “close over” invaginations that penetrate deeply into a cluster, and as such more robust methods such as concave hulls may need to be considered for some cellular structures [15].

## References:

1. Metropolis N, Ulam S. The Monte Carlo Method. J Am Stat Assoc. 1949;44: 335–341.

2. Preibisch S, Saalfeld S, Schindelin J, Tomancak P. Software for bead-based registration of selective plane illumination microscopy data. Nat Methods. Nature Publishing Group; 2010;7: 418–419. doi:10.1038/nmeth0610-418

3. Heit B, Kim H, Cosío G, Castaño D, Collins R, Lowell CA, et al. Multimolecular Signaling Complexes Enable Syk-Mediated Signaling of CD36 Internalization. Dev Cell. 2013; 1–12. doi:10.1016/j.devcel.2013.01.007

4. Díaz ME, Ayala G, León T, Zoncu R, Toomre D. Analyzing protein-protein spatial-temporal dependencies from image sequences using fuzzy temporal random sets. J Comput Biol. 2008;15: 1221–36. doi:10.1089/cmb.2008.0055

5. Cohn J. Theory of the radial distribution function. J Phys Chem. 1968;72: 608–616. doi:10.1021/j100848a037

6. Beale R, Pathria R, Beale P. Statistical Mechanics, 3rd Edition. Elsevier; 2011.

7. Kiskowski MA, Hancock JF, Kenworthy AK. On the use of Ripley’s K-function and its derivatives to analyze domain size. Biophys J. Biophysical Society; 2009;97: 1095–103. doi:10.1016/j.bpj.2009.05.039

8. Ester M, Kriegel H-P, Sander J, Xu X. A density-based algorithm for discovering clusters in large spatial databases with noise. Proc Second Int Conf Knowl Discov Data Min. 1996; 226–231. Available: http://citeseerx.ist.psu.edu/viewdoc/summary?doi=10.1.1.71.1980

9. Sander J, Ester M, Kriegel H-P, Xu X. Density-Based Clustering in Spatial Databases: The Algorithm GDBSCAN and Its Applications. Data Min Knowl Discov. 1998;2: 169–194. doi:10.1023/A:1009745219419

10. Ankerst M, Breunig MM, Kriegel H, Sander J. OPTICS: ordering points to identify the clustering structure. Proceedings of the 1999 ACM SIGMOD international conference on Management of data - SIGMOD ’99. New York, New York, USA: ACM Press; 1999. pp. 49–60. doi:10.1145/304182.304187

11. Daszykowski M, Walczak B, Massart DL. Looking for natural patterns in analytical data. 2. Tracing local density with OPTICS. J Chem Inf Comput Sci. 2002;42: 500–7. Available: http://www.ncbi.nlm.nih.gov/pubmed/12086507

12. Sander J, Qin X, Lu Z, Niu N, Kovarsky A. Automatic Extraction of Clusters from Hierarchical Clustering Representations. Adv Knowl Discov Data Min. 2003;2637: 75–87. doi:10.1007/3-540-36175-8_8

13. Hell SW, Wichmann J. Breaking the diffraction resolution limit by stimulated emission: stimulated-emission-depletion fluorescence microscopy. Opt Lett. 1994;19: 780–782. doi:10.1364/OL.19.000780

14. Heintzmann R, Jovin TM, Cremer C. Saturated patterned excitation microscopy--a concept for optical resolution improvement. J Opt Soc Am A Opt Image Sci Vis. 2002;19: 1599–1609. doi:10.1364/JOSAA.19.001599

15. Moreira A, Santos MY. Concave Hull: A K -Nearest Neighbours Approach for the Computation of the Region Occupied by a Set of Points. Iternational Conference on Computer Graphics Theory and Applications. INSTICC Press; 2007. pp. 61–68. Available: http://hdl.handle.net/1822/6429
